# Pivotal Roles for Ribonucleases in Streptococcus pneumoniae Pathogenesis

**DOI:** 10.1128/mBio.02385-21

**Published:** 2021-09-21

**Authors:** Dhriti Sinha, Jacob P. Frick, Kristen Clemons, Malcolm E. Winkler, Nicholas R. De Lay

**Affiliations:** a Department of Microbiology and Molecular Genetics, McGovern Medical School, University of Texas Health Science Center, Houston, Texas, USA; b Department of Biology, Indiana University Bloomington, Bloomington, Indiana, USA; c MD Anderson Cancer Center UTHealth Graduate School of Biomedical Sciences, University of Texas Health Science Center, Houston, Texas, USA; National Institute of Child Health and Human Development (NICHD)

**Keywords:** RNase Y, polynucleotide phosphorylase, posttranscriptional regulation, small RNAs

## Abstract

RNases perform indispensable functions in regulating gene expression in many bacterial pathogens by processing and/or degrading RNAs. Despite the pivotal role of RNases in regulating bacterial virulence factors, the functions of RNases have not yet been studied in the major human respiratory pathogen Streptococcus pneumoniae (pneumococcus). Here, we sought to determine the impact of two conserved RNases, the endoribonuclease RNase Y and exoribonuclease polynucleotide phosphorylase (PNPase), on the physiology and virulence of S. pneumoniae serotype 2 strain D39. We report that RNase Y and PNPase are essential for pneumococcal pathogenesis, as both deletion mutants showed strong attenuation of virulence in murine models of invasive pneumonia. Genome-wide transcriptomic analysis revealed that the abundances of nearly 200 mRNA transcripts were significantly increased, whereas those of several pneumococcal small regulatory RNAs (sRNAs), including the Ccn (CiaR-controlled noncoding RNA) sRNAs, were altered in the Δ*rny* mutant relative to the wild-type strain. Additionally, lack of RNase Y resulted in pleiotropic phenotypes that included defects in pneumococcal cell morphology and growth *in vitro*. In contrast, Δ*pnp* mutants showed no growth defect *in vitro* but differentially expressed a total of 40 transcripts, including the tryptophan biosynthesis operon genes and numerous 5′ *cis*-acting regulatory RNAs, a majority of which were previously shown to impact pneumococcal disease progression in mice using the serotype 4 strain TIGR4. Together, our data suggest that RNase Y exerts a global impact on pneumococcal physiology, while PNPase mediates virulence phenotypes, likely through sRNA regulation.

## INTRODUCTION

The Gram-positive bacterium Streptococcus pneumoniae (pneumococcus) is a common colonizer of the human nasopharynx, where it can remain as a commensal. However, specific signals, including host viral infection and environmental and nutritional stress, can stimulate S. pneumoniae to disperse into other host tissues (1, 2), including the lungs, blood, and brain, and dissemination of pneumococcus into these tissues leads to pneumonia, sepsis, and meningitis, respectively. Pneumococcal infections result in over 1 million deaths annually worldwide (3). S. pneumoniae has been shown in murine infection models to have distinct gene expression profiles depending on whether it resides in the blood, brain, nasopharynx, or lungs (4, 5), indicating that it has to adapt to the different conditions in these tissues to survive. Furthermore, pneumococcus also rapidly reprograms its gene expression pattern upon exposure to host cells, such as macrophages (6) and lung epithelial cells (7, 8). To rapidly adapt to changes in their environment, bacteria not only need to modulate the transcription of particular genes but also must turn over existing small regulatory RNAs (sRNAs) or mRNAs that encode proteins detrimental under the new set of conditions. RNases control the steady-state levels and turnover of various classes of RNAs (9, 10). In the model Gram-positive bacterium Bacillus subtilis, the primary RNase responsible for initiating RNA decay was shown to be RNase Y (11). Depletion of this RNase from B. subtilis impacted expression of ∼20% of all open reading frames in its genome (12) and led to a 2-fold increase in the half-life of bulk mRNA (11).

RNase Y is the functional but evolutionarily distinct equivalent of endoribonuclease RNase E of Gram-negative bacteria. RNase Y consists of an N-terminal transmembrane domain followed by a coiled-coil domain, an RNA-binding KH domain, a catalytic HD domain, and a C-terminal domain (12). Like RNase E, RNase Y associates with the membrane (13) and also serves as the organizing component of the RNA degradosome, the central RNA-degrading machine in bacteria (12). RNase Y forms this complex by interacting with the RNA helicase CshA, the RNases J1 and J2, the glycolytic enzymes phosphofructokinase and enolase, and the exoribonuclease PNPase (12, 14). The dual-function RNases J1/J2 exhibit both endonucleolytic and 5′-to-3′ exoribonucleolytic activities and are unique to Gram-positive bacteria (15). In Bacillus subtilis and Streptococcus pyogenes, the decay intermediates resulting from endonucleolytic cleavage are primarily cleared by PNPase, which functions as the major 3′-to-5′ exoribonuclease (16, 17). PNPase has also been shown to significantly impact global mRNA turnover under cold stress in B. subtilis and Staphylococcus aureus, similar to what has been demonstrated in Escherichia coli (18, 19).

Very recent transcriptome sequencing (RNA-seq) studies in S. pyogenes have uncovered the RNA targetomes of both RNase Y and PNPase and further demonstrated that these two proteins work in concert to regulate 5′-regulatory RNA turnover and the stability of polycistronic mRNAs (20). These results are consistent with the previously implicated role of RNase Y in mediating decay of 5′ *cis*-acting regulatory RNAs (*S*-adenosylmethionine, T-box, and riboflavin riboswitches) in S. aureus and B. subtilis (11, 21, 22). Interestingly, results of a recent study that globally examined protein-RNA associations in S. pneumoniae via gradient profiling by sequencing (Grad-seq) indicate that PNPase interacts with several small RNAs *in vivo* (23). However, compared to those in Gram-negative bacteria, the detailed mechanisms by which major RNases in Gram-positive organisms, such as RNase Y and PNPase, impact sRNA-dependent regulation and *trans*-acting sRNA levels remain largely unknown; however, there has been some evidence for RNase Y-dependent turnover of sRNAs (e.g., RsaA in S. aureus and RoxS in B. subtilis [24]). Independent studies have further indicated an indirect role of RNase Y in regulating the abundance of two other *trans*-acting sRNAs, VR-RNA and FasX, in the important Gram-positive pathogens Clostridium perfringens and S. pyogenes, respectively (25, 26). The extents to which RNase Y orthologs from different species contribute to growth and RNA decay vary considerably (22). These findings further emphasize that various Gram-positive organisms, including pathogens, may employ different mechanistic strategies to mediate RNA decay and processing.

In spite of the crucial roles of RNases in impacting bacterial stress response by altering gene expression, we do not know about the functions of major pneumococcal RNases. In the present work, we report characterization of two conserved RNases, RNase Y and PNPase, in S. pneumoniae serotype 2 strain D39. We demonstrate that RNase Y functions as a broadly pleiotropic regulator whose absence significantly impacts the pneumococcal mRNA transcriptome, growth, virulence, and stability and function of conserved pneumococcal Ccn (CiaR-controlled noncoding RNA) sRNAs. In contrast, PNPase impacts the abundance of several important transcripts, including riboswitches that were previously implicated in pneumococcal virulence control. The absence of PNPase consistently resulted in a strong virulence defect *in vivo* while displaying no obvious phenotypes *in vitro*. Together, our work has uncovered for the first time the crucial roles of two well-conserved RNases in regulating pneumococcal physiology and virulence.

## RESULTS

### RNase Y is required for normal pneumococcal growth and cell morphology.

**FIG 1:**
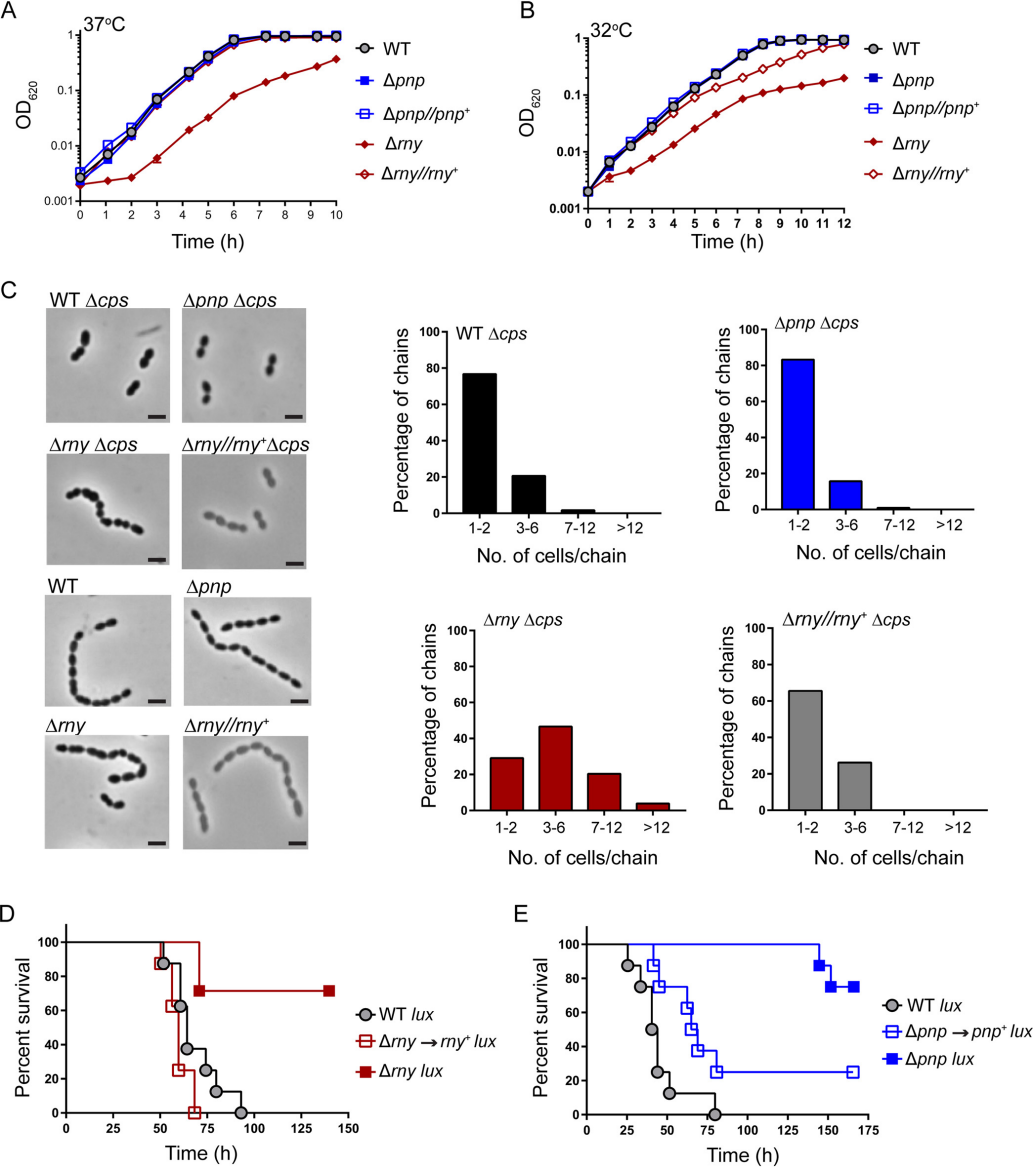
Phenotypes of Δ*rny* and Δ*pnp* mutants. (A and B) Growth characteristics of the encapsulated D39 parent strain (IU1781), isogenic Δ*rny* (IU4599) and Δ*pnp* (IU4883) strains, and complemented Δ*rny*//*rny*^+^ (IU4834) and Δ*pnp*//*pnp*^+^ (IU5510) strains, grown statically at 37°C and at 32°C in 5-day-old BHI broth in an atmosphere of 5% CO_2_. Growth curves represent data from three independent replicates for each strain at 37°C or 32°C. Average growth rates and growth yields are listed in Table S2. (C) Representative phase-contrast images of the D39 wild-type strain (WT; IU1781), its derived Δ*cps* (IU1824), Δ*rny* (NRD10092), Δ*pnp* (IU4883), Δ*cps* Δ*rny* (NRD10109), and Δ*cps* Δ*pnp* (NRD10108) mutants, and Δ*rny*//*rny*+ (NRD10388) and Δ*cps* Δ*rny*//*rny*+ (NRD10389) complemented strains in early exponential growth phase. Distributions of chain lengths were based on 100 to 200 chains from at least two independent cultures of each strain. Bars, 2 μm. (D and E) Survival curve analysis showing disease progression in an invasive murine model of pneumonia. ICR male mice were inoculated intranasally with ∼10^7^ CFU in 50-μl inocula of the D39 parent expressing a *lux* luminescence cassette (D39 Tn*4001 luxABCDE* [IU1918]) or isogenic mutants (Δ*rny* Tn*4001 luxABCDE* [IU6838]; Δ*pnp* Tn*4001 luxABCDE* [IU6622]; *rny*^+^ Tn*4001 luxABCDE* [IU7152]; and *pnp*^+^ Tn*4001 luxABCDE* [IU7154]). Eight animals were infected per strain, and disease progression was followed in real time by survival curve analysis (see Materials and Methods). Survival curves were analyzed by Kaplan-Meier statistics and log-rank tests to determine *P* values.

Prior studies showed that deletion of *rny*, the gene encoding RNase Y, from B. subtilis and C. perfringens caused a drastic reduction in growth, but the effect of removal of this gene on S. pyogenes and S. aureus growth was modest (25, 27–29). However, deletion of *pnp* led to a cold-sensitive phenotype in B. subtilis, similar to what was observed for Escherichia coli (30, 31). Therefore, we assessed the effects of clean deletion in *rny* or *pnp* (Table S1) on pneumococcal growth at both optimal (37°C) and lower (32°C) temperatures. We found that at 37°C in brain heart infusion (BHI) broth, the Δ*rny* mutant exhibited a significant reduction in growth rate and yield compared to the wild-type (WT) strain (Fig. 1A; Table S2). The average doubling time and growth yield for the Δ*rny* mutant were ∼69 min and 0.37, compared with ∼39 min and 0.96 for the WT strain. The observed growth defect of the Δ*rny* mutant was restored by expressing *rny* from a constitutive P*_mal_*_(c)_ promoter at the ectopic CEP (chromosomal expression platform) locus or by repairing the mutation to the WT allele at the native locus (Fig. 1A; Fig. S1A to D; Table S2). We also observed that the growth deficiency of the Δ*rny* mutant became more pronounced in 15- and 25-day-old BHI compared to freshly prepared (≤5-day-old) BHI, whereas the isogenic parental strain grew similarly in fresh and aged BHI (Fig. S1 to D; Table S2). In contrast, the Δ*pnp* mutant grew like the WT strain in BHI broth at 37°C (Fig. 1A; Fig. S1A to D). In contrast to E. coli and B. subtilis (30, 31), the pneumococcal Δ*pnp* mutant did not show a cold-sensitive (CS) phenotype (Fig. 1B) at 32°C, the lowest temperature at which S. pneumoniae D39 grows well. Finally, the Δ*rny* mutant was not cold sensitive, as the growth rate differences between the WT and Δ*rny* mutant were not significantly different at 32°C compared to 37°C (Fig. 1B; Fig. S1E to G; Table S2).

10.1128/mBio.02385-21.1 TABLE S1 S. pneumoniae strains and primers used in this study. Download Table S1, XLSX file, 0.02 MB. Copyright © 2021 Sinha et al. 2021 Sinha et al. https://creativecommons.org/licenses/by/4.0/ This content is distributed under the terms of the Creative Commons Attribution 4.0 International license.

10.1128/mBio.02385-21.2 TABLE S2 Growth characteristics of Δ*rny* mutants used in this study. Download Table S2, XLSX file, 0.01 MB. Copyright © 2021 Sinha et al. 2021 Sinha et al. https://creativecommons.org/licenses/by/4.0/ This content is distributed under the terms of the Creative Commons Attribution 4.0 International license.

10.1128/mBio.02385-21.5 FIG S1 Growth curves of Δ*rny* and Δ*pnp* mutants in batches of BHI broth of different ages at optimal (37°C) and lower (32°C) temperatures. Growth curves of the encapsulated D39 parent strain (IU1781) and isogenic Δ*rny* (NRD10092, IU4599), Δ*pnp* (IU4883), Δ*rny→rny^+^* (NRD10305; *rny* repair strain), Δ*pnp→pnp^+^* (NRD10303; *pnp* repair strain), Δ*rny*/*rny^+^* (IU4834; *rny*-complemented strain), and Δ*pnp*/*pnp^+^* (IU5510; *pnp* complemented strain) strains in 5-day-old (A), 15-day-old (B, D), or 25-day-old (C) BHI broth at 37°C and 3-day-old (E) and 15-day-old (F, G) BHI broth at 32°C (E to G). Points and error bars (where not visible, error bars are smaller than the symbol) represent the means and SEM of the growth curves for three independent replicates for each strain tested. Average growth rates and growth yields are listed in Table S2. Download FIG S1, TIF file, 1.6 MB. Copyright © 2021 Sinha et al. 2021 Sinha et al. https://creativecommons.org/licenses/by/4.0/ This content is distributed under the terms of the Creative Commons Attribution 4.0 International license.

To gain insight into the growth impairment of Δ*rny* mutants, we examined cells from early-exponential-phase cultures (optical density at 620 nm [OD_620_] ≈ 0.1 to 0.15) of the WT and Δ*rny* mutant by phase-contrast microscopy. We found that the Δ*rny* mutant exhibits significant morphological defects. Occasionally, Δ*rny* mutants formed minicells at the ends or in the middle of a chain, indicating a possible cell division defect (Fig. 1C). The abnormalities in cell morphology that we observed in the encapsulated Δ*rny* mutant were even more pronounced in a Δ*cps* mutant lacking capsule (Fig. 1C). This observation is consistent with previous findings that capsule tends to dampen pneumococcal cell shape and division phenotypes (32). In addition, the Δ*rny* Δ*cps* mutant formed longer chains comprising 4 to 12 cells/chain, in contrast to the WT parent, which occurred mainly as diplococci (Fig. 1C). The observed chaining effect of the Δ*rny* mutant was reversed by expressing *rny* from a constitutive P*_mal_*_(c)_ promoter at the ectopic CEP locus (Fig. 1C). Finally, we did not observe any morphological differences between the Δ*pnp* mutant and the WT parent in either the *cps*^+^ or the Δ*cps* background (Fig. 1C). We conclude that RNase Y, but not PNPase, is required for S. pneumoniae D39 normal growth and cell morphology.

### Lack of RNase Y or PNPase attenuates S. pneumoniae D39 pathogenesis.

Lack of RNase Y in S. pyogenes and S. aureus resulted in virulence attenuation (28, 33, 34). Therefore, we determined the consequences of the *rny* and *pnp* deletions on S. pneumoniae D39 pathogenesis using a murine invasive pneumonia model (see Materials and Methods). Both the Δ*rny* and Δ*pnp* mutants were substantially attenuated for virulence compared to the WT parent (Fig. 1D and E). Of the mice inoculated with the Δ*pnp* or Δ*rny* mutant, 75% and 87%, respectively, survived the course of the experiment (∼170 h), whereas the median survival time for the WT parent strain ranged from 42 h to 64 h (Fig. 1D and E; Fig. S2A). To determine if the attenuated virulence observed in each case was correlated with loss of *rny* or *pnp* function, we repaired Δ*rny* or Δ*pnp* back to the *rny*^+^ or *pnp*^+^ allele, respectively, by allelic exchange (see Materials and Methods). The *rny*^+^ and *pnp^+^* repaired strains displayed median survival times of 60 h and 67 h, respectively, indicative of full virulence. Taking these results together, we conclude that both RNase Y and PNPase contribute to pneumococcal pathogenesis.

10.1128/mBio.02385-21.6 FIG S2 Additional phenotypes of Δ*rny* and Δ*pnp* mutants. (A) Survival curve analysis showing disease progression of Δ*pnp* mutant compared to a WT parent in a murine pneumonia model. Eight ICR male mice were inoculated intranasally with ∼10^7^ CFU in 50-μl inocula of the D39 parent (IU1781) strain or an isogenic Δ*pnp* mutant (IU4883), and disease progression was followed in real time by a survival curve analysis (see Materials and Methods). Survival curves were analyzed by Kaplan-Meier statistics and log-rank tests to determine *P* values. (B) Determination of transformation frequency of the Δ*rny* mutant compared to a WT parent in a spontaneous transformation assay. Percentage of spontaneous transformants and the corresponding transformation frequencies were determined for the D39 parent (D39 Δ*cps*; IU1824) and its derived isogenic Δ*rny* mutant (Δ*cps* Δ*rny*; NRD10109) (see Materials and Methods). Data and error bars represent the means and SEM from at least five independent experiments. Download FIG S2, TIF file, 0.3 MB. Copyright © 2021 Sinha et al. 2021 Sinha et al. https://creativecommons.org/licenses/by/4.0/ This content is distributed under the terms of the Creative Commons Attribution 4.0 International license.

### RNase Y and PNPase impact the pneumococcal mRNA transcriptome differently.

**FIG 2:**
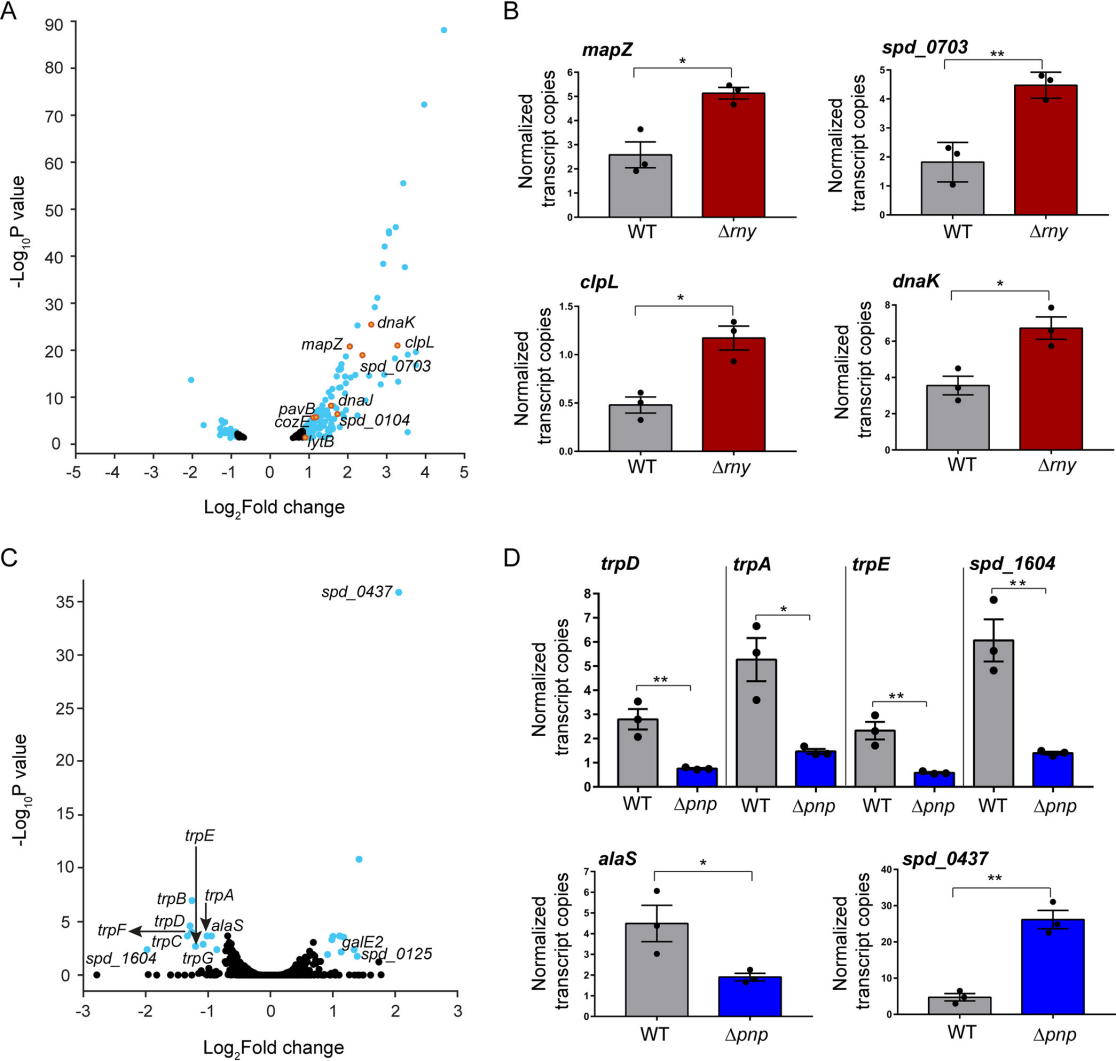
Impact of RNase Y and PNPase on mRNA transcriptome of S. pneumoniae D39. (A and C) Volcano plots showing genome-wide changes in mRNA transcript levels in a Δ*rny* mutant (A) and a Δ*pnp* mutant (C) relative to the D39 parent strain. RNA was extracted from exponentially growing cultures of the WT D39 parent (IU3116) and isogenic Δ*rny* (IU5504) and Δ*pnp* (IU5498) mutants in triplicate and analyzed by mRNA-seq as described in Materials and Methods. Orange and cyan dots represent genes with relative transcript changes of >1.8-fold as the cutoff (log_2_ fold change = 0.85), with an adjusted *P* value cutoff of <0.05. Relative transcript level changes of genes below the cutoff values are considered insignificant and are in black. The *x* axis represents gene fold changes, and the *y* axis represents corresponding *P* values plotted on a logarithmic scale. mRNAs that were significantly upregulated or downregulated in the Δ*rny* mutant or Δ*pnp* mutant compared to the parent are listed in Tables 1 and 2, respectively. (B and D) ddPCR analysis was used to determine copy numbers of indicated transcripts in a wild-type D39 parent (WT; IU1781) and isogenic mutants (Δ*rny*, NRD10092; Δ*pnp*, IU4883). Transcript numbers were normalized to the 16S transcript number, which served as the internal control. Bars and error bars represent the means and standard errors of the means (SEM) from at least three independent experiments. *, *P* < 0.05; **, *P* < 0.01.

To identify target transcripts of RNase Y or PNPase that influence pneumococcal physiology, next, we compared the genome-wide transcriptome profiles of Δ*rny* or Δ*pnp* mutant relative to the WT parent grown in matched batches of BHI broth at 37°C in an atmosphere of 5% CO_2_ using mRNA-sequencing (mRNA-seq) analysis (see Materials and Methods). mRNA-seq of the Δ*rny* mutant revealed that 185 transcripts were significantly upregulated compared to the WT parent strain, using a cutoff of a >1.8-fold change and a *P* value adjusted for multiple testing (*P*_adj_) of <0.05. In contrast, only 28 genes were significantly downregulated in the Δ*rny* mutant compared to the WT strain (Table 1; Fig. 2A). The upregulated transcripts encode proteins that are involved in diverse cellular functions, including translation; transcription; transport and metabolism of carbohydrates, amino acids, nucleotides, coenzymes, and inorganic ions; cell wall and envelope biogenesis; and stress response (Table 1). In particular, several transcripts that were upregulated in the Δ*rny* mutant are under the regulatory control of the WalRK, LiaFSR, PnpRS, or CiaRH two-component system (TCS) (Table 1). Notably, relative transcript abundance for genes encoding important cell division and cell wall proteins, including *mapZ*, *cozE*, *gpsB*, *lytB*, *licB*, *licC*, *licA*, *tarI*, *tarJ*, *spd_0703*, and *spd_0104*, were increased by ∼2-5-fold in the Δ*rny* strain. Lack of RNase Y also increased the relative expression of genes involved in stress response (*clpL*, 9-fold; *dnaK*, 6-fold; *dnaJ*, 3-fold; *hptX*, 2-fold) and *pavB* (∼2-fold), which encodes a fibronectin-binding protein involved in pneumococcal virulence.

**TABLE 1:** Genes showing changes in relative mRNA transcript amounts in a Δ*rny* mutant compared to the *rny*^+^ parent strain during exponential growth in BHI broth^*a*^

*spd* gene tag	Gene	Known or predicted function	Fold change	*P*_adj_ value
Increased relative expression				
SPD_0026	scRNA	Protein and peptide secretion	2.00	8.35E−04
SPD_0056^*b*^	*vanZ*	Teicoplanin resistance protein VanZ	2.11	4.27E−03
SPD_0057^*b*^	*purH*	Bifunctional purine biosynthesis protein PurH	1.97	1.26E−03
SPD_0064	*cpsR*	Transcriptional regulator, GntR family protein	1.98	4.90E−05
SPD_0072		Glyoxalase family protein	1.88	5.35E−03
**SPD_0080**^*c*^	***pavB***	**Fibronectin-binding protein PavB**	**2.27**	**1.78E−06**
SPD_0084		IS630-Spn1, transposase Orf1	3.04	2.63E−04
SPD_0085		Hypothetical protein	2.66	1.52E−05
SPD_0086		Hypothetical protein	3.03	1.75E−12
SPD_0087		Hypothetical protein	2.99	7.64E−11
SPD_0090^*c*^		ABC transporter, substrate-binding protein	2.67	3.66E−02
**SPD_0104**^*d*^^,^^*e*^		**Aggregation-promoting factor, LysM domain protein**	**3.32**	**3.99E−07**
SPD_0105		Hypothetical protein	2.03	1.65E−02
SPD_0159		Membrane protein putative	1.88	6.33E−04
SPD_0187	*nrdD*	Ribonucleotide reductase of class III, large subunit	3.86	1.06E−13
SPD_0188		Hypothetical protein	2.97	7.33E−11
SPD_0189		Acetyltransferase, GNAT family protein	2.52	1.50E−08
SPD_0190	*nrdG*	Ribonucleotide reductase of class III, activating protein	2.45	2.25E−07
SPD_0191		Hypothetical protein	2.26	4.24E−06
SPD_0232^*c*^		Cellobiose-specific PTS IIA component	1.90	4.04E−02
SPD_0280	*celR*	Transcriptional antiterminator of lichenan operon, BglG family protein	2.04	4.87E−02
SPD_0283	*celD*	Cellobiose-specific PTS IIC component	2.82	4.52E−07
SPD_0289^*c*^	*eda*	Bifunctional 4-hydroxy-2-oxoglutarate aldolase/2-deydro-3-deoxyphosphogluconate aldolase	3.09	7.69E−09
SPD_0290^*c*^		2-Dehydro-3-deoxygluconokinase	3.42	9.63E−13
SPD_0291^*c*^		Putative 4-deoxy-l-threo-5-hexosulose-uronate ketol-isomerase	2.90	7.85E−04
SPD_0292^*c*^		2-Deoxy-d-gluconate 3-dehydrogenase	2.95	1.99E−04
SPD_0295^*c*^		Hyaluronate-oligosaccharide-specific PTS IIB component	2.42	1.25E−02
SPD_0296^*c*^		Hyaluronate-oligosaccharide-specific PTS IIC component	2.97	4.70E−05
SPD_0297^*c*^		Hyaluronate-oligosaccharide-specific PTS IID component	2.85	5.77E−04
**SPD_0308**	***clpL***	**ATP-dependent Clp proteinase ATP-binding subunit ClpL**	**9.68**	**9.97E−22**
**SPD_0339**	***gpsB***	**Cell division protein GpsB**	**1.88**	**2.51E−02**
SPD_0341	*rlmL*	23S rRNA methyltransferase	4.75	5.59E−26
**SPD_0342**	***mapZ***	**Midcell-anchored-protein Z**	**4.13**	**1.65E−21**
SPD_0345	*cbpF*	Choline-binding protein CbpF	1.89	1.42E−04
SPD_0373	*mip*	Macrophage infectivity potentiator protein	2.15	2.04E−03
SPD_0380^*c*^	*fabH*	3-Oxoacyl-(acyl carrier protein) synthase FabH	1.90	1.40E−04
SPD_0437^*b*^	*ribU*	Substrate-specific component RibU of riboflavin ECF transporter	22.2	7.74E−89
SPD_0438		Membrane-associated phospholipid phosphatase	8.31	4.97E−46
SPD_0439		Hypothetical protein	8.33	1.41E−45
SPD_0440		Hypothetical protein	7.70	8.61E−43
SPD_0441	*rpoE*	DNA-directed RNA polymerase delta subunit	3.49	5.59E−17
SPD_0443	*nptA*	Sodium-dependent phosphate transporter NptA	2.85	8.90E−12
SPD_0452	*creX*	Integrase/recombinase CreX	2.06	4.12E−02
**SPD_0460**^*c*^^,^^*f*^	***dnaK***	**Chaperone protein DnaK**	**6.07**	**3.52E−26**
**SPD_0461**^*c*^^,^^*f*^	***dnaJ***	**Chaperone protein DnaJ**	**2.96**	**6.49E−09**
SPD_0501	*bglG*	Beta-glucoside *bgl* operon antiterminator BglG	13.4	2.46E−20
SPD_0502	*bglF*	Beta-glucoside-specific IIBCA components	11.0	2.14E−38
SPD_0503	*bglA-2*	6-Phospho-beta-glucosidase	7.51	4.26E−39
SPD_0522	*vex2*	ABC transporter ATP-binding protein Vex2	1.82	1.82E−03
SPD_0523	*vex3*	Peptide ABC transporter membrane-spanning permease Vex3	1.85	9.48E−04
SPD_0540^*c*^		Cysteine ABC transporter substrate-binding protein	2.27	2.68E−06
SPD_0550	*rplK*	Ribosomal protein L11	3.39	1.56E−16
SPD_0551	*rplA*	Ribosomal protein L1	3.57	9.49E−18
SPD_0608	*pyrF*	Orotidine 5′-phosphate decarboxylase	2.18	2.39E−06
SPD_0609	*pyrE*	Orotate phosphoribosyltransferase	2.05	4.85E−05
SPD_0616^*c*^	*glnQ3*	Glutamine ABC transporter ATP-binding protein GlnQ3	11.6	9.01E−20
SPD_0617^*c*^	*glnP3b*	Glutamine ABC transporter permease GlnP3b	9.80	5.02E−14
SPD_0618^*c*^	*glnP3a*	Glutamine ABC transporter permease GlnP3a	9.27	5.48E−19
SPD_0627	*ykoC*	Transmembrane component YkoC of energizing module of thiamin-regulated ECF transporter for hydroxymethylpyrimidine	1.82	8.02E−03
SPD_0628	*tenA*	Thiaminase II TenA	1.88	8.85E−04
SPD_0629	*thiW*	Substrate-specific component ThiW of putative thiazole ECF transporter	1.86	4.65E−03
SPD_0676		Hypothetical protein	2.54	5.46E−09
SPD_0677		Hypothetical protein	2.32	2.25E−07
SPD_0678	*rimM*	16S rRNA processing protein RimM	2.30	1.55E−07
SPD_0679	*trmD*	tRNA (guanine-N1)-methyltransferase	2.36	5.40E−08
SPD_0680		Anaerobic ribonucleoside-triphosphate reductase	2.09	6.79E−06
SPD_0681		Hypothetical protein	3.63	3.84E−07
SPD_0683		Hypothetical protein	1.87	1.63E−03
SPD_0692		Hypothetical protein	1.89	4.36E−04
**SPD_0703**^*d*^		**Hypothetical protein**	**5.18**	**1.15E−19**
SPD_0716		IS630-Spn1, transposase Orf1	1.85	1.70E−02
**SPD_0768**	***cozE***	**Coordinator of zonal cell elongation**	**2.16**	**2.16E−06**
SPD_0775^*c*^^,^^*g*^		Acetyltransferase	2.81	1.33E−03
SPD_0803^*f*^		Putative phage shock protein C	2.60	1.24E−04
SPD_0806		Hypothetical protein	2.18	5.09E−04
SPD_0852	*pyrDb*	Dihydroorotate dehydrogenase, catalytic subunit	1.84	1.85E−03
**SPD_0853**^*d*^	***lytB***	**Endo-beta-*N*-acetylglucosaminidase LytB**	**1.87**	**3.88E−02**
SPD_0872^*b*^		Membrane protein, putative	1.84	1.23E−03
SPD_0882		IS630-Spn1, transposase Orf2, truncation	2.76	6.41E−08
SPD_0883		Hypothetical protein	3.80	1.58E−11
SPD_0884		Hypothetical protein	4.20	6.64E−15
SPD_0898		Membrane protein, putative	2.28	1.50E−07
SPD_0913^*g*^^,^^*h*^		Extracellular protein	3.06	6.56E−04
SPD_0930	*pezA*	Antitoxin PezA	2.46	9.80E−05
SPD_0931	*pezT*	Bifunctional UDP-N-acetylglucosamine kinase/zeta toxin PezT	2.27	1.01E−05
SPD_0932		Hypothetical protein	1.90	4.08E−03
SPD_0933		Hypothetical protein	2.18	2.07E−05
SPD_0940	*rffD*	UDP-*N*-acetyl-d-mannosaminuronic acid dehydrogenase	2.15	3.07E−02
SPD_0954		Hypothetical protein	1.98	4.54E−05
SPD_0995		Membrane protein, putative	2.09	1.14E−05
SPD_1004^*b*^	*gapN*	Glyceraldehyde-3-phosphate dehydrogenase, NADP dependent	1.90	2.03E−03
SPD_1014		IS630-Spn1, transposase Orf1	1.90	2.78E−02
SPD_1023	*xerS*	Tyrosine recombinase XerS	1.85	1.10E−03
SPD_1045		Hypothetical protein	11.6	2.73E−03
SPD_1046	*lacG-2*	6-Phospho-β-galactosidase	7.18	1.92E−13
SPD_1047^*c*^	*lacE-2*	Lactose-specific PTS IIBC components	7.64	1.65E−15
SPD_1048^*c*^	*lacF-2*	Lactose-specific PTS IIA component	5.84	2.69E−15
SPD_1049	*lacT*	Transcriptional antiterminator LacT	4.75	7.81E−07
SPD_1073		Bifunctional *O*-acetylhomoserine sulfhydrylase/*O*-succinylhomoserine sulfhydrylase	1.88	2.86E−03
SPD_1075	*nirC*	Formate-nitrate transporter	2.15	6.55E−06
SPD_1090	*panT*	Substrate-specific component PanT of putative pantothenate ECF transporter	1.80	1.86E−03
**SPD_1123**^*g*^	***licC***	**Cholinephosphate cytidylyltransferase LicC**	**1.88**	**2.50E−04**
**SPD_1124**^*g*^	***licB***	**Choline permease LicB**	**1.92**	**1.69E−04**
**SPD_1125**^*g*^	***licA***	**Choline kinase LicA**	**2.04**	**1.42E−05**
**SPD_1126**^*g*^	***tarJ***	**Ribulose-5-phosphate reductase**	**2.09**	**7.78E−06**
**SPD_1127**^*g*^	***tarI***	**Ribitol-5-phosphate cytidylyltransferase**	**2.10**	**7.83E−06**
**SPD_1138**	***hptX***	**Heat shock protein HtpX**	**2.04**	**1.25E−05**
SPD_1139	*lemA*	LemA protein	2.08	1.44E−05
SPD_1148	*rplS*	Ribosomal protein L19	6.76	7.46E−32
SPD_1159		Hypothetical protein	2.58	4.76E−07
**SPD_1160**^*f*^		**ABC transporter ATP-binding protein**	**2.68**	**3.74E−10**
SPD_1161		Hypothetical protein	2.36	2.91E−04
SPD_1175		Hypothetical protein	2.18	9.50E−05
SPD_1176^*c*^		ABC transporter ATP-binding protein	2.26	6.98E−06
SPD_1177^*c*^		ABC transporter ATP-binding/membrane spanning protein	2.65	2.09E−07
SPD_1178	*ptrB*	Protease II	2.75	7.36E−07
SPD_1179	*lanL*	Lanthionine biosynthesis protein LanL	3.26	2.40E−08
SPD_1267		ABC transporter, ATP-binding protein	2.88	1.10E−02
SPD_1294		Conserved hypothetical protein	1.99	1.64E−03
SPD_1295^*b*^		Hemolysin		2.11E−03
SPD_1296	*pdx2*	Glutamine amidotransferase, SNO family protein, putative	2.28	4.89E−04
SPD_1297	*pdx1*	Pyridoxine biosynthesis protein	2.18	2.78E−04
SPD_1301		NADPH-dependent FMN reductase	1.96	5.26E−03
SPD_1302^*b*^		Oxidoreductase, putative	1.90	6.25E−04
SPD_1355		Conserved hypothetical protein	2.33	2.59E−04
SPD_1357^*c*^	*aliB*	Oligopeptide ABC transporter, oligopeptide-binding protein AliB	2.53	1.95E−07
SPD_1377		Conserved hypothetical protein	2.42	4.77E−06
SPD_1402		Non-heme iron-containing ferritin	5.47	4.86E−10
SPD_1439	*rpsO*	Ribosomal protein S15	6.46	7.13E−30
SPD_1535	*scrR*	Sucrose operon repressor	1.89	2.54E−04
SPD_1577		Conserved hypothetical protein	3.86	2.13E−19
SPD_1592		Acetyltransferase, GNAT family protein	2.00	1.32E−03
SPD_1594		Transcriptional regulator	2.24	2.75E−06
SPD_1595		Conserved hypothetical protein	2.52	8.83E−07
SPD_1603		Conserved hypothetical protein	3.13	4.15E−06
SPD_1604		Conserved hypothetical protein	3.81	3.98E−15
SPD_1614^*c*^	*phoU2*	Phosphate transport system regulatory protein PhoU, putative	1.88	3.49E−02
SPD_1633	*galT-2*	Galactose-1-phosphate uridylyltransferase	4.57	1.84E−15
SPD_1634	*galK*	Galactokinase	3.73	1.59E−06
SPD_1635	*galR*	Galactose operon repressor	2.46	3.17E−07
SPD_1640	*pnuC*	Nicotinamide mononucleotide transporter PnuC, putative	9.39	6.74E−47
SPD_1649^*c*^	*piuB*	Iron compound ABC transporter, permease protein	2.00	3.22E−04
SPD_1650^*c*^	*piuC*	Iron compound ABC transporter, permease protein	1.93	2.58E−04
SPD_1651^*b*^^,^^*c*^	*piuD*	Iron compound ABC transporter, ATP-binding protein	1.98	1.40E−04
SPD_1665^*c*^	*treR*	Trehalose operon repressor	2.18	2.53E−06
SPD_1673^*c*^	*gtfA*	Sucrose phosphorylase	1.89	1.01E−03
SPD_1676^*c*^	*rafF*	Sugar ABC transporter, permease protein	2.89	2.85E−06
SPD_1677^*c*^	*rafE*	Sugar ABC transporter, sugar-binding protein	2.76	1.19E−07
SPD_1678	*aga*	Alpha-galactosidase AgaN	3.51	8.12E−13
SPD_1679	*msmR*	*msm* operon regulatory protein MsmR	2.05	4.96E−05
SPD_1680		Biotin–acetyl-CoA-carboxylase ligase	2.19	2.04E−05
SPD_1707		Conserved hypothetical protein	13.5	1.50E−17
SPD_1716		Conserved hypothetical protein	1.94	4.20E−03
SPD_1717		Membrane protein, putative	1.81	1.07E−02
SPD_1719		PAP2 family protein	1.80	8.27E−03
SPD_1720		Conserved hypothetical protein	2.10	1.44E−03
SPD_1830	*bguA*	Glycosyl hydrolase, family protein 1	2.18	5.95E−06
SPD_1831^*c*^	*bguD*	PTS system, IIC component	2.27	1.41E−06
SPD_1832^*c*^	*bguB*	PTS system, IIB component	2.08	2.76E−04
SPD_1833^*c*^	*bguC*	PTS system, IIA component	2.40	4.46E−07
SPD_1865		Putative Zn-dependent alcohol dehydrogenase	2.12	7.51E−05
SPD_1868	*tgt*	tRNA-guanine transglycosylase	2.05	1.34E−05
SPD_1899		Glutamine amidotransferase, class 1	2.45	3.28E−04
**SPD_1910**^*h*^	***pstS1***	**Phosphate ABC transporter periplasmic-phosphate-binding protein PstS1**	**3.87**	**5.51E−08**
**SPD_1911**^*h*^	***pstC1***	**Phosphate transport system permease protein PstC1**	**2.87**	**2.97E−03**
**SPD_1912**^*h*^	***pstA1***	**Phosphate transport system permease protein PstA1**	**2.77**	**1.37E−02**
**SPD_1913**^*h*^	***pstB1***	**Phosphate transport ATP-binding protein PstB1**	**2.64**	**2.23E−02**
**SPD_1914**^*h*^	***phoU1***	**Phosphate transport system regulatory protein PhoU1**	**2.96**	**1.81E−04**
SPD_1932^*c*^	*malP*	Maltodextrin phosphorylase	2.47	4.54E−05
SPD_1933^*c*^^,^^*h*^	*malQ*	4-Alpha-glucanotransferase	1.87	3.33E−02
SPD_1962^*c*^		Membrane protease family protein	2.18	9.75E−06
SPD_1976^*c*^	*arcB*	Ornithine carbamoyltransferase	4.15	1.57E−21
SPD_1977^*c*^	*arcC*	Carbamate kinase	3.28	3.79E−15
SPD_1978^*c*^	*arcD*	Arginine/ornithine antiporter ArcD	3.48	1.43E−16
SPD_1979^*c*^		Putative Xaa-His dipeptidase	3.55	8.53E−17
SPD_1984	*ybbK*	Putative membrane protease subunit YbbK	2.42	4.36E−04
SPD_1989		Mannose-specific PTS IID component	3.46	2.29E−04
SPD_1996	*fucR*	l-Fucose operon activator	1.93	1.23E−04
SPD_2007		Macrolide-efflux protein	2.63	4.89E−07
SPD_2033	*hpf*	Ribosomal hibernation promotion factor	2.10	1.86E−03
SPD_2037	*cysK*	Cysteine synthase	1.85	2.71E−02
SPD_2041	*tsf*	Translation elongation factor Ts	10.7	3.00E−56
SPD_2042	*rpsB*	Ribosomal protein S2	15.6	5.46E−73

Decreased relative expression				
SPD_0214		Adenylate kinase	0.25	2.12E−14
SPD_0383^*c*^	*fabD*	Malonyl CoA-acyl carrier protein transacylase	0.54	2.06E−03
SPD_0390^*c*^	*accA*	Acetyl-CoA carboxylase, carboxyl transferase, alpha subunit	0.55	6.08E−03
SPD_0447		Transcriptional regulator, MerR family protein	0.49	1.79E−03
SPD_0448		Glutamine synthetase, type I	0.44	2.05E−03
SPD_0449		Conserved hypothetical protein	0.31	8.77E−05
SPD_0451		Type I restriction-modification system, S subunit, putative	0.45	9.10E−04
SPD_0518		Conserved hypothetical protein	0.49	5.19E−04
SPD_0519		Conserved hypothetical protein	0.51	1.36E−02
SPD_0520		Transposase, putative, truncation	0.43	3.75E−05
SPD_0674		Ribosomal protein S16	0.54	2.36E−03
SPD_0675		Conserved hypothetical protein	0.55	5.79E−03
SPD_1410		tRNA-Leu	0.49	4.64E−02
SPD_1683		tRNA-Ile	0.52	4.39E−03
SPD_1691		tRNA-Arg	0.49	9.77E−04
SPD_1789		Cell wall surface anchor family protein	0.45	1.99E−05
SPD_1801		ABC transporter, ATP-binding protein	0.41	4.40E−04
SPD_1802		Conserved hypothetical protein	0.42	8.26E−06
SPD_1803		Conserved hypothetical protein	0.45	4.57E−03
SPD_1879		tRNA-Leu	0.47	4.14E−03
SPD_1881		tRNA-His	0.55	2.74E−02
SPD_2011^*c*^		Glycerol uptake facilitator protein	0.41	1.25E−02
SPD_2012^*c*^		Alpha-glycerophosphate oxidase	0.42	1.30E−03
SPD_2013^*c*^		Glycerol kinase	0.42	2.75E−03

a RNA extraction and mRNA-seq analyses were performed as described in Materials and Methods. RNA was prepared from cultures of isogenic strains IU3116 (wild-type parent; D39 *rpsL1 rny^+^* CEP::P_c_-[Kan^r^-*rpsL*^+^]) and IU5504 (D39 *rpsL1* Δ*rny* CEP::P_c_-[Kan^r^-*rpsL*^+^]) (Table S1). Fold changes (1.8-fold cutoff) and adjusted *P* values (*P* < 0.05) are based on three independent biological replicates. Boldface indicates genes mentioned in the text.

b Member of the CbpRS two-component system regulon (82).

c Member of the SaeRS two-component system regulon (83).

d Member of the WalRK two-component system regulon (55).

e Member of the TCS07/YesMN two-component system regulon (84).

f Member of the LiaFSR two-component system regulon (58).

g Member of the CiaRH two-component system regulon (85).

h Member of the PnpRS two-component system regulon (67).

Deletion of *pnp* had substantially less impact on relative mRNA transcript amounts, with significant changes in abundance of only 20 transcripts (Table 2; Fig. 2C). Interestingly, a majority of mRNA transcripts that were differentially regulated in the Δ*pnp* mutant were shown by a previous transposon insertion sequencing (Tn-seq) screen of serotype 4 strain TIGR4 to be important for colonization of the nasopharynx and/or infection of the lungs in murine infection models (35) (Table 2). In particular, the relative abundance of transcripts corresponding to the tryptophan biosynthesis operon (*trpABCDEFG*), including the upstream gene *spd_1604*, were maximally downregulated by ∼3- to 4-fold. In addition, the relative transcript amount of *alaS*, which encodes alanyl-tRNA-synthetase, was downregulated by ∼2-fold in the Δ*pnp* mutant compared to the WT strain (Table 2; Fig. 2C).

**TABLE 2:** Genes showing changes in relative mRNA transcript amounts in a Δ*pnp* mutant compared to the *pnp*^+^ parent strain during exponential growth in BHI broth^*a*^

*spd* tag no.	Gene	Known or predicted function	Fold change	*P*_adj_ value
Increased relative expression				
SPD_0125		Hypothetical protein	2.62	1.72E−02
SPD_0437^*b*^	*ribU*	Substrate-specific component RibU of riboflavin ECF transporter	4.16	1.33E−36
SPD_0771^*b*^	*fruR*	Transcriptional repressor of the fructose operon	2.00	2.33E−04
SPD_0975^*b*^	*radC*	DNA repair protein RadC	2.00	4.75E−04
SPD_1579		Hypothetical protein	2.53	4.13E−03
SPD_1586^*b*^		Multiple sugar metabolism operon regulatory protein	2.68	1.39E−11
SPD_1588		Hypothetical protein	1.89	1.18E−02
SPD_1612^*b*^	*galE-2*	UDP-glucose 4-epimerase	2.19	6.74E−03
SPD_1707		Hypothetical protein	2.25	2.79E−04
SPD_1792		Hypothetical protein	2.16	2.13E−04

Decreased relative expression				
SPD_1216^*c*^	*alaS*	Alanyl-tRNA synthetase	0.52	2.13E−04
SPD_1596^*b*^^,^^*d*^	*trpA*	Tryptophan synthase alpha chain	0.49	2.13E−04
SPD_1597^*d*^	*trpB*	Tryptophan synthase beta chain	0.42	1.03E−07
SPD_1598^*d*^	*trpF*	Phosphoribosylanthranilate isomerase	0.42	9.06E−05
SPD_1599^*b*^^,^^*d*^	*trpC*	Indole-3-glycerol phosphate synthase	0.40	2.13E−04
SPD_1600^*b*^^,^^*d*^	*trpD*	Anthranilate phosphoribosyltransferase	0.41	2.42E−05
SPD_1601^*b*^^,^^*d*^	*trpG*	Bifuncational anthranilate synthase	0.44	1.97E−03
SPD_1602^*b*^^,^^*d*^	*trpE*	Anthranilate synthase, amidase component	0.47	1.29E−03
SPD_1604		Hypothetical protein	0.25	4.06E−03
SPD_2012	*glpO*	Alpha-glycerophosphate oxidase	0.55	4.06E−03

a RNA extraction and mRNA-seq analyses were performed as described in Materials and Methods. RNA was prepared from cultures of isogenic strains IU3116 (wild-type parent; D39 *rpsL1 pnp^+^* CEP::P_c_-[Kan^r^-*rpsL*^+^]) and IU5498 (D39 *rpsL1* Δ*pnp* CEP::P_c_-[Kan^r^-*rpsL*^+^]) (Table S1). Fold changes (1.8-fold cutoff) and adjusted *P* values (*P* < 0.05) are based on three independent biological replicates.

b Role in virulence according to Tn-seq studies in TIGR4 (36).

c Likely essential gene according to Tn-seq studies in TIGR4 (36).

d Member of the tryptophan (*trp*) biosynthesis operon.

Results from mRNA-seq analyses were confirmed by reverse transcriptase droplet digital PCR (RT-ddPCR) as described in Materials and Methods. Consistent with the RNA-seq results, the relative transcript amounts of *mapZ* (∼2-fold), *spd_0703* (∼3-fold), *clpL* (∼4-fold), and *dnaK* (∼2-fold) increased in the Δ*rny* mutant compared to WT strain (Fig. 2B). In the Δ*pnp* mutant, RT-ddPCR showed that the relative transcript amounts of *spd_1604*-*trpD*-*trpA*-*trpE* and *alaS* decreased by ∼4-fold and 2.4-fold, respectively, whereas the relative amount of *spd_0437* (*ribU*) increased by ∼6-fold (Fig. 2D), again consistent with the RNA-seq results. Together, these data confirm the relative changes in steady-state mRNA transcript amounts caused by lack of RNase Y or PNPase in S. pneumoniae.

### RNase Y and PNPase mediate the sRNA transcriptome of S. pneumoniae.

Previous studies demonstrate that RNase Y directly and indirectly impacts sRNA levels in several important Gram-positive pathogens (25, 36, 37), whereas PNPase promotes the stability of some sRNAs and degrade others in E. coli (38, 39). A recent Grad-seq study indicates that S. pneumoniae PNPase binds to several sRNAs, including CcnA, CcnB, CcnC, CcnD, and Spd_sr34 (23). To further understand how RNases modulate the stability and function of sRNAs expressed by S. pneumoniae D39, we sought to identify the sRNAs targeted by RNase Y and PNPase using sRNA sequencing (sRNA-seq) (see Materials and Methods). At least 112 distinct sRNAs have been identified in S. pneumoniae D39 (40–45).

**FIG 3:**
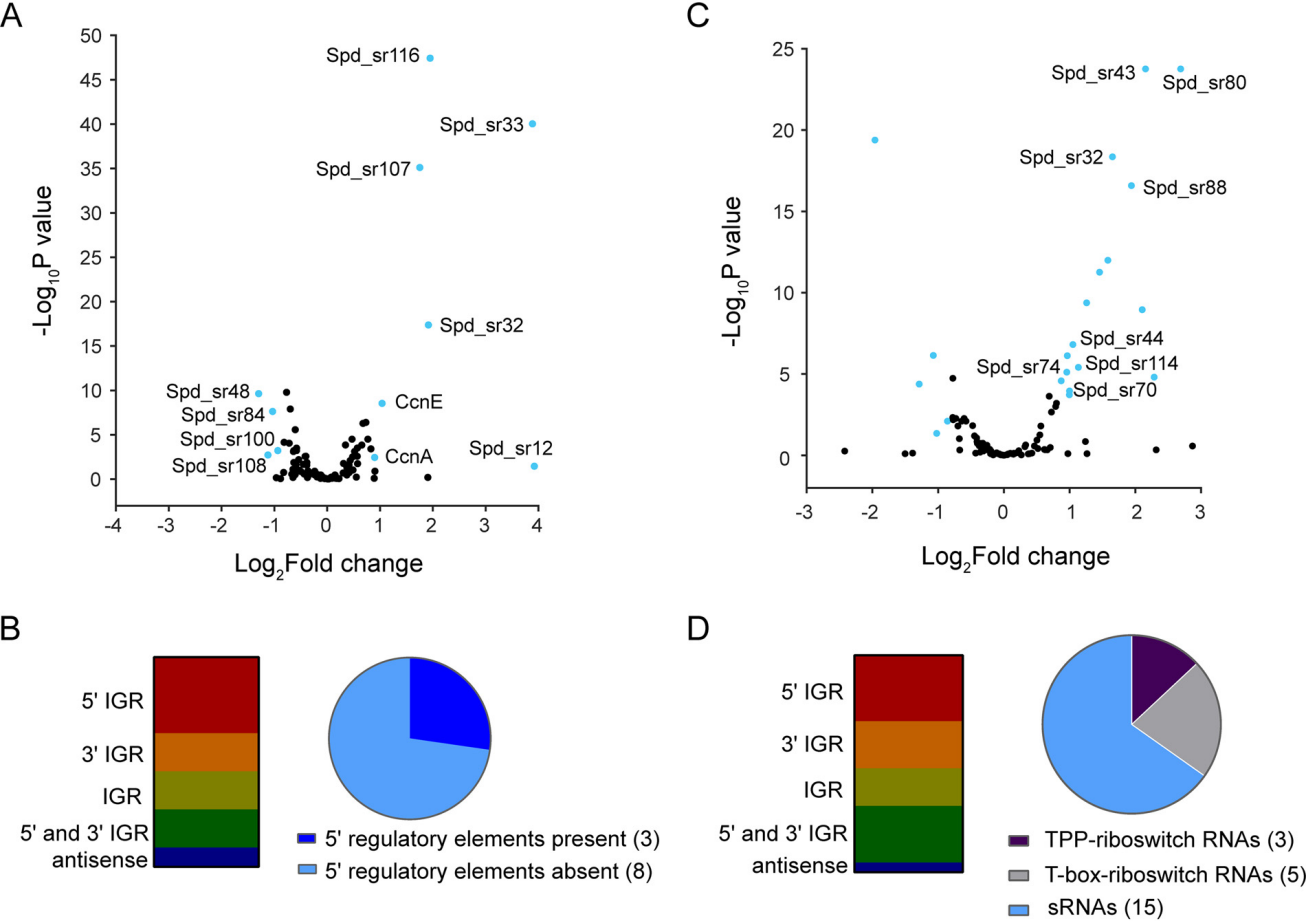
Impact of RNase Y and PNPase on sRNA transcriptome of S. pneumoniae D39. (A and C) Volcano plot showing genome-wide changes in sRNA transcript levels in a Δ*rny* mutant (A) or Δ*pnp* mutant (C) relative to the D39 parent strain. RNA was extracted from exponentially growing cultures of the WT D39 parent (IU1781) and isogenic Δ*rny* (NRD10092) and Δ*pnp* (IU4883) mutants in triplicate and analyzed by sRNA-seq analysis as described in Materials and Methods. Cyan dots represent genes with relative transcript changes of >1.8-fold as the cutoff (log_2_ fold change = 0.85), with an adjusted *P* value cutoff of <0.05. Relative transcript level changes of genes below the cutoff values are considered insignificant and are in black. The *x* axis represents gene-fold changes, and the *y* axis represents corresponding *P* values plotted on a logarithmic scale. sRNAs that were significantly upregulated or downregulated in the Δ*rny* mutant or Δ*pnp* mutant compared to the parent are listed in Tables 3 and 4, respectively. (B and D) Distribution of sRNAs that were differentially regulated in a Δ*rny* mutant (B) or a Δ*pnp* (D) mutant compared to the parent in different genomic contexts as described previously (40). Pie chart graphs indicate the percent distribution of the sRNAs based on the presence or absence of 5′ *cis*-regulatory elements in their sequence. IGR, intergenic region.

**FIG 4:**
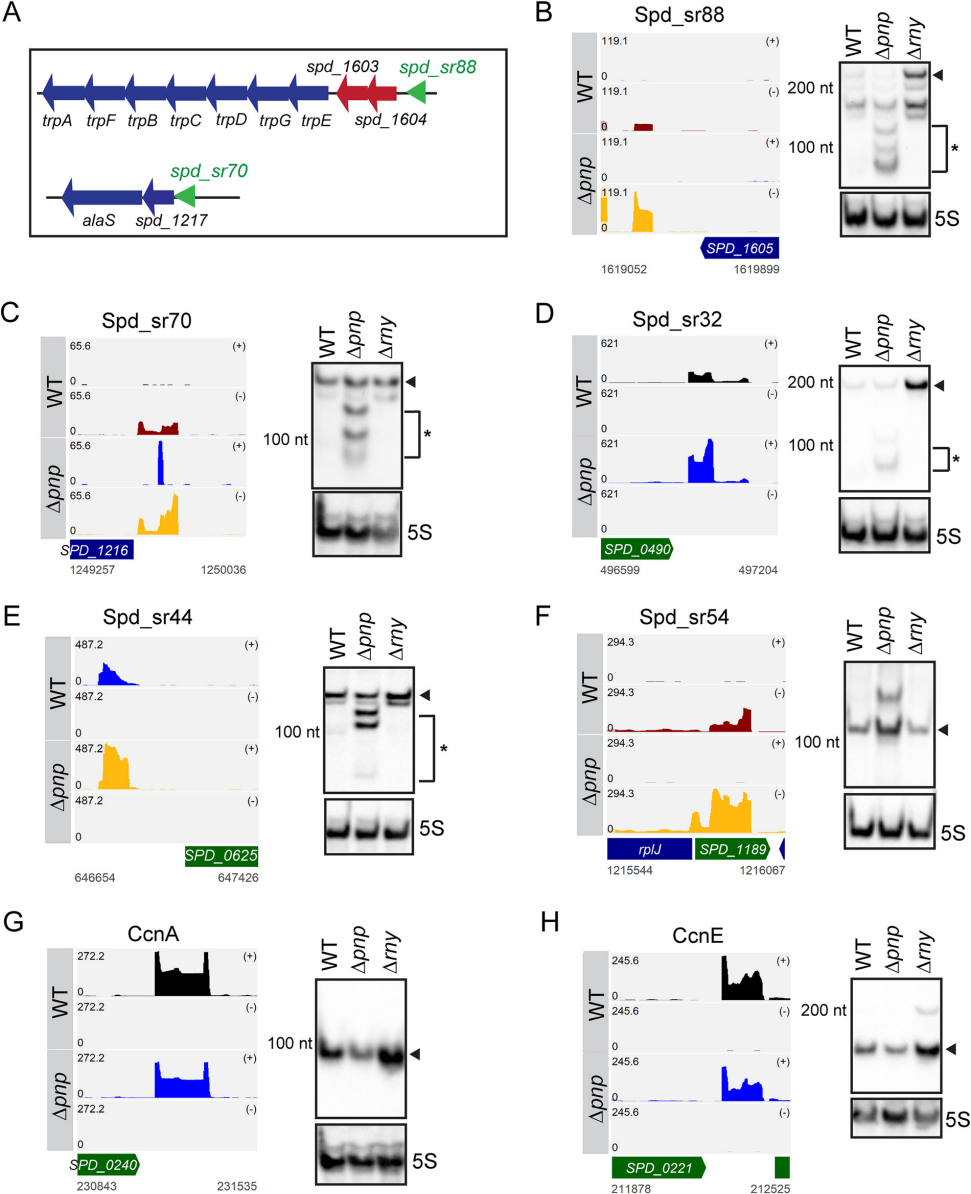
PNPase plays an important role in the decay and processing of riboswitch RNAs in S. pneumoniae D39. (A) Genetic context of two T-box riboswitches, Spd-sr88 and Spd-sr70, in S. pneumoniae D39. (B to H) Read coverage maps of a subset of sRNAs and their flanking regions that were differentially regulated in a Δ*pnp* mutant (IU4883) compared to the WT parent (IU1781) in sRNA-seq. Coverage represents depth per million reads of paired-end sRNA fragments and was averaged between normalized replicates (see Materials and Methods). In each coverage graph, open reading frames (ORFs) encoded on the plus or minus strand are in green or blue, respectively. Northern blots detecting the sRNAs are presented alongside the read coverage maps. Black triangles and asterisks indicate the full-length sRNA transcripts and sRNA decay products, respectively. Corresponding coverage maps for the sRNAs presented in panels B to H in a Δ*rny* mutant (NRD10092) compared to the WT parent (IU1781) are presented in Fig. S4. Quantification of signal intensity for each full-length sRNA normalized to 5S rRNA amount is displayed in Fig. S5, and the probes used are listed in Table S3.

sRNA-seq analysis revealed that 11 sRNAs (∼10% of total sRNAs) showed a >1.8-fold change in relative amount between the Δ*rny* mutant and WT strain (Table 3; Fig. 3A). Seven sRNAs were upregulated in the Δ*rny* mutant compared to the WT strain, whereas only 4 were downregulated. The putative regulatory RNAs impacted by Δ*rny* fall into all five categories of sRNAs based on their location relative to previously annotated genes in D39 (Fig. 3B). Three of the sRNAs differentially expressed in the Δ*rny* mutant contain regulatory elements; Spd-sr12 and Spd-sr32 contain T-box riboswitches, and Spd-sr48 has an L20 leader sequence that regulates the expression of downstream ribosomal genes. Interestingly, among the significantly upregulated sRNAs in the Δ*rny* mutant are 2 Ccn sRNAs (CcnA and CcnE) (Table 3; Fig. 3A), which are among the five homologous, highly conserved intergenic pneumococcal sRNAs under positive transcriptional control of the CiaR response regulator and function to inhibit competence development via base-pairing with the precursor of the competence stimulatory peptide-encoding mRNA *comC* (86, 87). Seven of 11 sRNAs that were differentially expressed in the Δ*rny* mutant relative to the WT strain were experimentally validated using Northern blotting. We found that four sRNAs (CcnE, CcnA, Spd-sr12, and Spd-sr32) are significantly upregulated, while for the sRNAs Spd-sr100 and Spd-sr116, the annotated full-length transcripts could not be detected in the Δ*rny* mutant (Fig. 4; Fig. S3 and S5). However, we did observe that a higher-molecular-weight band corresponding to Spd-sr116 was increased in abundance only in a Δ*rny* mutant (Fig. S3). Spd-sr108 was the only sRNA for which we observed a significant difference in abundance between the Δ*rny* mutant and WT strain by RNA-seq but not by Northern blotting analysis (Table 3; Fig. S3 and S5). In addition to these sRNAs, we probed for 12 additional sRNAs that were not significantly differentially expressed in the Δ*rny* mutant relative to the WT strain in the RNA-seq analysis. Northern blots revealed that eight of these sRNAs (Spd-sr43, Spd-sr44, Spd-sr73, Spd-sr74, Spd-sr80, Spd-sr83, Spd-sr88, and Spd-sr114) were upregulated in the Δ*rny* mutant relative to the wild-type strain, whereas 4 others (Spd-sr70, Spd-sr54, Spd-sr82, and Spd-sr96) were unaffected by Δ*rny* (Fig. 4; Fig. S3 and S5). Together, these data confirm that the cellular amounts of a relatively small number of sRNAs are changed in the Δ*rny* mutant.

**TABLE 3:** Relative sRNA transcript level changes in strain a Δ*rny* mutant compared to the *rny*^+^ parent strain during exponential growth in BHI broth^*a*^

sRNA ID	Flanking genes	Fold change	*P*_adj_
Increased relative expression			
**CcnE**	*spd_0221*, *spd_0222*	2.05	2.87E−09
**CcnA**	*spd_0240*, *ruvB*	1.86	3.56E−03
**SPD_SR32**^*b*^	*spd_0490*, *spd_0491*	3.77	4.24E−18
SPD_SR33	*spd_0500*, *licT*	14.8	9.22E−41
**SPD_SR12**^*b*^	*ppC*, *spd_0954*	15.2	3.46E−02
SPD_SR107	*malP*, *spd_1931*	3.37	7.75E−36
**SPD_SR116**	*spd_2043*, *rpsB*	3.86	3.73E−48

Decreased relative expression			
SPD_SR48^*b*^	*spd_0846*, *infC*	−2.46	2.31E−10
SPD_SR84	*spd_1578*, *spd_1577*	−2.05	2.36E−08
**SPD_SR100**	*pbp2A*, *secE*	−1.92	6.02E−03
**SPD_SR108**^*c*^	*spd_1939*, *malR*	−2.18	0.0018

a RNA extraction and sRNA-seq analyses were performed as described in Materials and Methods. RNA was prepared from cultures of the encapsulated parent strain IU1781 (wild-type parent; D39 *rpsL1 rny^+^*) and its derived mutant NRD10092 (D39 *rpsL1* Δ*rny*) (Table S1). Fold changes (1.8-fold cutoff) and *P* values (*P*_adj_ < 0.05) are based on three independent biological replicates. sRNAs validated in this study are in bold (Fig. 4; Fig. S3 and S5).

b 5′ regulatory element present.

c Spd-sr108 levels were comparable between the wild type and a Δ*rny* mutant on Northern blots (Fig. S5).

10.1128/mBio.02385-21.7 FIG S3 Northern blot validations for sRNA-seq analysis. (A) Northern blot validations for sRNAs that showed differential expression in a Δ*rny* mutant (NRD10092) relative to the WT parent (IU1781) parent in sRNA-seq analysis (Table 3). Changes in Spd-sr20 amount were not detected by sRNA-seq (Table 3). The levels of the sRNAs were also determined in an isogenic Δ*pnp* mutant (IU4883). (B) Northern blot validations for sRNAs that showed differential expression in a Δ*pnp* mutant relative to the WT parent (IU1781) in sRNA-seq analysis (Table 4). Changes in Spd-sr96 amount were not detected by RNA-seq analysis (Table 4), but its levels were still examined by Northern blotting. The levels of the sRNAs were also determined in an isogenic Δ*rny* mutant. Black triangles and asterisks indicate the full-length sRNA transcripts and sRNA decay products, respectively. Download FIG S3, TIF file, 2.3 MB. Copyright © 2021 Sinha et al. 2021 Sinha et al. https://creativecommons.org/licenses/by/4.0/ This content is distributed under the terms of the Creative Commons Attribution 4.0 International license.

10.1128/mBio.02385-21.9 FIG S5 Corresponding Northern blot quantifications for Fig. 4 and Fig. S3. sRNA steady-state levels were determined from Northern blots of RNA isolated from exponentially growing cultures of the Δ*pnp* mutant (IU4883), Δ*rny* mutant (NRD10092) or WT parent (IU1781) strain as described in Materials and Methods. Signal intensities of the full-length sRNA transcripts were quantified using Northern blotting and normalized to their corresponding loading controls (5S rRNA). The normalized value for each sRNA isolated from the WT strain was set at 100, and levels of sRNA in each mutant are scaled to this value. The full-length transcript for Spd-sr100 was detected only in a Δ*pnp* mutant (Fig. S3). Download FIG S5, TIF file, 1.5 MB. Copyright © 2021 Sinha et al. 2021 Sinha et al. https://creativecommons.org/licenses/by/4.0/ This content is distributed under the terms of the Creative Commons Attribution 4.0 International license.

10.1128/mBio.02385-21.3 TABLE S3 Droplet digital PCR primers and oligonucleotide probes used in this study. Download Table S3, XLSX file, 0.01 MB. Copyright © 2021 Sinha et al. 2021 Sinha et al. https://creativecommons.org/licenses/by/4.0/ This content is distributed under the terms of the Creative Commons Attribution 4.0 International license.

10.1128/mBio.02385-21.8 FIG S4 Read coverage maps for sRNAs shown in Fig. 4 in the Δ*rny* mutant compared to the WT parent. Tracks labeled WT and Δ*rny* correspond to the read coverages for the sRNAs and their flanking regions in the WT (IU1781) and Δ*rny* (NRD10092) mutant strain, respectively, obtained from sRNA-seq. Coverage represents depth per million reads of paired-end sRNA fragments from averaged normalized replicates (see Materials and Methods). In each coverage graph, ORFs on the plus and the minus strands are color coded in green and blue, respectively. Download FIG S4, TIF file, 0.5 MB. Copyright © 2021 Sinha et al. 2021 Sinha et al. https://creativecommons.org/licenses/by/4.0/ This content is distributed under the terms of the Creative Commons Attribution 4.0 International license.

In contrast to the Δ*rny* mutant, 21% of the pneumococcal sRNA transcriptome was significantly altered in the Δ*pnp* mutant. Twenty-three sRNAs exhibited >1.8-fold differences in relative expression in the Δ*pnp* mutant (Table 4; Fig. 3C), where 17 and 6 sRNAs were significantly up- and downregulated, respectively. Notably, approximately half of the sRNAs upregulated in a Δ*pnp* mutant relative to the WT strain are riboswitch RNAs. Spd-sr32, Spd-sr70, Spd-sr74, Spd-sr80, and Spd-sr88 are characterized by the presence of a T-box riboswitch, while Spd-sr43, Spd-sr44, and Spd-sr114 contain a thiamine pyrophosphate (TPP) riboswitch element (Table 4; Fig. 3C and D). The riboswitch RNAs Spd-sr44 and Spd-sr88 are particularly interesting, because Tn-seq screens with the serotype 4 strain (TIGR4) of S. pneumoniae indicated that the loss of *spd-sr44* or *spd-sr88* results in reduced pneumococcal fitness in murine blood and lung infection, respectively (46). 5′-intergenic and 3′-intergenic sRNAs are in the overrepresented category of sRNAs that showed differential regulation in Δ*pnp* compared to the WT strain (Fig. 3D). Finally, we validated the expression of a total of 14 of 23 sRNAs that were significantly differentially expressed in the Δ*pnp* mutant relative to the WT strain (Fig. 4; Fig. S3 and S5). Taken together, these data suggest that both RNase Y and PNPase play important roles in regulating the relative amounts of different sets of pneumococcal regulatory RNAs.

**TABLE 4:** Relative sRNA transcript level changes in a Δ*pnp* mutant compared to the *pnp*^+^ parent strain during exponential growth in BHI broth^*a*^

sRNA ID	Flanking genes	Fold change	*P*_adj_
Increased relative expression			
**SPD_SR32**^*b*^	*spd_0490*, *spd_0491*	3.14	4.39E−19
SPD_SR33	*spd_0500*, *licT*	4.88	1.55E−05
**SPD_SR43**^*c*^	*lctO*, *spd_0622*	4.45	1.74E−24
**SPD_SR44**^*c*^	*thiE1*, *spd_0625*	2.07	1.54E−07
SPD_SR57	*spd_0988*, *spd_0987*	1.83	2.62E−05
**SPD_SR54**	*spd_1190*, *rplJ*	1.95	7.51E−07
**SPD_SR70**^*b*^	*spd_1216*, *spd_1217*	2.00	0.00011
**SPD_SR73**	*spd_1289*, *spd_1288*	2.39	4.19E−10
**SPD_SR74**^*b*^	*spd_1308*, *spd_1307*	1.94	7.70E−06
SPD_SR77	*asnS*, *rpsF*	2.00	0.00019
**SPD_SR80**^*b*^	*spd_1441*, *spd_1442*	6.45	1.74E−24
SPD_SR81	*spd_1448*, *spd_1447*	4.30	1.11E−09
**SPD_SR82**	*spd_1455*, *spd_1454*	2.74	5.50E−12
**SPD_SR88**^*b*^	*spd_1605*, *spd_1604*	3.84	2.60E−17
**SPD_SR100**	*pbp2A*, *secE*	17.9	5.51E−75
**SPD_SR114**^*c*^	*cbpD*, *spd_2027*	2.19	3.88E−06
**SPD_SR116**	*spd_2043*, *rpsB*	2.99	1.01E−12

Decreased relative expression			
**CcnA**^*d*^	*spd_0240*, *ruvB*	−2.03	4.46E−02
SPD_SR36	*metF*, *pnp*	−15.9	1.36E−60
SPD_SR61	*spd_1080*, *spd_1079*	−2.11	7.19E−07
**SPD_SR83**	*recG*, *spd_1506*	−3.90	4.12E−20
SPD_SR95	*dinF*, *lytA*	−2.44	4.17E−05
SPD_SR101	*spd_1834*, *spd_1833*	−1.81	7.93E−03

a RNA extraction and sRNA-seq analyses were performed as described in Materials and Methods. RNA was prepared from cultures of the encapsulated parent strain IU1781 (wild-type parent; D39 *rpsL1 pnp^+^*) and its derived mutant IU4883 (D39 *rpsL1* Δ*pnp*) (Table S1). Fold changes (1.8-fold cutoff) and *P* values (*P*_adj_ < 0.05) are based on three independent biological replicates. sRNAs validated in this study are in bold (Fig. 4; Fig. S3 and S5).

b 5′ regulatory element and T-box element present.

c 5′ regulatory element and TPP riboswitch element present.

d CcnA sRNA levels were comparable between the wild type and the Δ*pnp* mutant on Northern blots (Fig. S5).

### PNPase and RNase Y play important roles in riboswitch RNA decay and processing.

T-box-containing riboswitch RNAs that are upregulated in the Δ*pnp* mutant include Spd-sr88 and Spd-sr70, which are located within the 5′ untranslated regions (UTRs) of the *trp* operon (encoding enzymes involved in tryptophan biosynthesis) and *alaS* (encoding alanyl-tRNA synthetase) operon, respectively (Table 4; Fig. 4A). Northern blotting confirmed increases in *spd-sr88* and *spd-sr70* in the Δ*pnp* mutant compared to the WT strain determined by RNA-seq analysis (Table 4) and showed accumulations of decay products of these sRNAs (Fig. 4B and C). Concurrently, relative transcript amounts of both *alaS* and the entire *trp* operon, including the upstream gene *spd_1604*, are decreased by ∼2-fold and ∼2- to 4-fold, respectively, in the Δ*pnp* mutant in mRNA-seq analysis (Table 2; Fig. 3C and D). Based on these observations, we further tested the expression profiles of six other TPP or T-box riboswitch RNAs that also showed increased relative expression in the Δ*pnp* mutant (Table 4). We observed a similar pattern of accumulation of decay intermediates for Spd-sr32, Spd-sr43, Spd-sr44, Spd-sr74, Spd-sr80, and Spd-sr114 in the Δ*pnp* mutant, but not in the WT or Δ*rny* strain (Fig. 4D and E; Fig. S3). In addition, we noticed that the full-length transcripts for all six of these riboswitch RNAs were more abundant in a Δ*rny* mutant than that in a WT strain (Fig. 4B and D; Fig. S3 and S4). While Spd-sr32 was identified by sRNA-seq analysis to be significantly upregulated by ∼4-fold in the Δ*rny* mutant compared to WT (Table 3), the increased relative transcript steady-state levels of the other riboswitch RNAs (Spd-sr43, Spd-Sr44, Spd-sr74, Spd-sr88, and Spd-sr114) in a Δ*rny* mutant were not detected by this transcriptomics-based approach but were detected independently by Northern blot analysis (Fig. S3). Taking these results together, we conclude that both RNase Y and PNPase jointly function in the processing and decay of riboswitch regulatory RNAs.

### RNase Y regulates Ccn sRNA stability and function.

**FIG 5:**
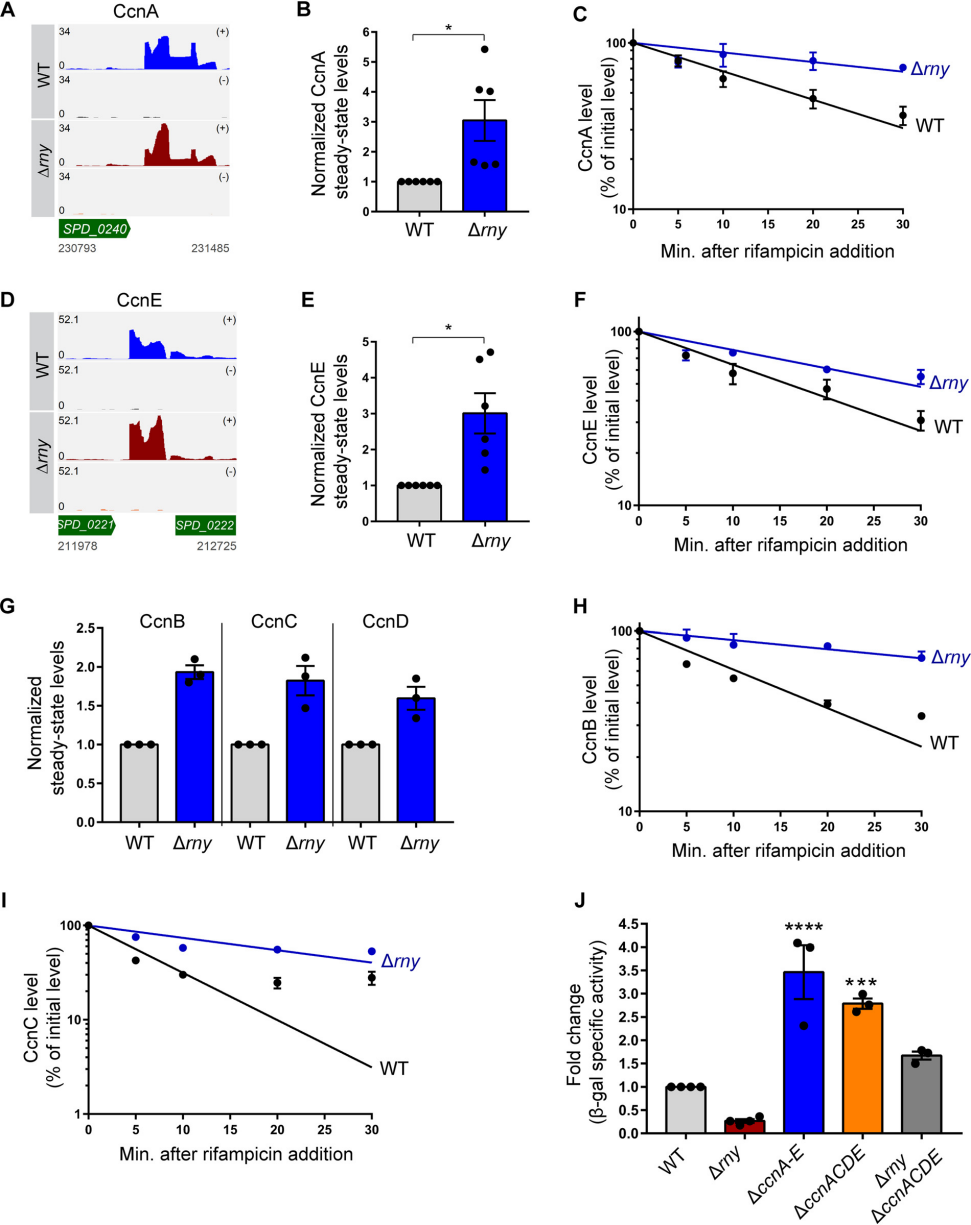
RNase Y regulates Ccn sRNA stability and function in S. pneumoniae D39. (A and D) Read coverage maps of CcnA and CcnE in a Δ*rny* mutant (NRD10092) compared to the WT parent (IU1781). Track labels corresponding to read coverage maps are described in the legend to Fig. 4. (B, E, and G) CcnA, CcnE, CcnB, CcnC, and CcnD steady-state levels were determined on Northern blots following extraction of RNA from exponentially growing cultures of a Δ*rny* mutant (NRD10092) and a WT parent strain (IU1781) as described in Materials and Methods. Signal intensities in the Northern blots were quantified and normalized to 5S RNA amount. (C, F, H, and I) RNA amount time course experiment to determine the intrinsic stability of CcnA, CcnE, CcnB, and CcnC in a Δ*rny* mutant (NRD10092) and the WT strain (IU1781) after treatment with rifampin to stop transcription, as described in Materials and Methods. Semilog sRNA decay curves were generated by fitting the normalized signal intensities determined on Northern blots for each time point sample. Points and error bars in the curves (where not visible, error bars are smaller than the symbol) represent the means and SEM from at least three independent experiments. sRNA half-life measurements corresponding to RNA stability curves are listed in Table S4. (J) β-Galactosidase assay to determine the impact of RNase Y on Ccn sRNA-mediated *comC* translational regulation. Expression of the *comC*'-'*lacZ* translational fusion was monitored by β-galactosidase assays of samples removed from exponentially growing cultures of the unencapsulated D39 parent strain (NRD10041) and isogenic Δ*cps* Δ*rny comC*'-'*lacZ* (NRD10113), Δ*cps* Δ*ccnA*–*E comC*'-'*lacZ* (NRD10187), Δ*cps* Δ*ccnACDE comC*'-'*lacZ* (NRD10054), and Δ*cps* Δ*rny* Δ*ccnACDE comC*'-'*lacZ* (NRD10120) mutants. Bars and error bars represent means and SEM from at least three independent experiments. *, *P* < 0.05; **, *P* < 0.01; ***, *P* < 0.001; ns, not significant.

After validating by Northern blotting, the ∼3-fold increases in relative steady-state levels of CcnA and CcnE in the Δ*rny* mutant (Fig. 4G and H; Fig. 5A, B, D, and E; Fig. S6) in accordance with our sRNA-seq data (Table 3), we sought to further define the mechanism by which RNase Y regulates of the abundance of the Ccn sRNAs in S. pneumoniae D39. Therefore, we measured the stability of CcnA and CcnE in exponentially growing cultures of a Δ*rny* mutant or a WT strain after blocking transcription initiation by adding rifampin (Fig. 5C and F). The relative half-life (*t*_1/2_) of CcnA increased by approximately 3-fold in the Δ*rny* mutant compared to the WT (*t*_1/2_ = 52.2 min versus 17.6 min [Fig. 5C]), while that of CcnE increased by ∼2-fold (*t*_1/2_ = 28.4 min versus 15.8 min [Fig. 5F]) (Table S4). These findings prompted us to test the impact of RNase Y on the stability and the corresponding steady-state levels of the remaining three Ccn sRNAs. The relative transcript levels for CcnB, CcnC, and CcnD, were similarly upregulated by ∼2-fold in the Δ*rny* mutant compared to WT (Fig. 5G). Consistent with these increased amounts, CcnB and CcnC were significantly stabilized in the Δ*rny* mutant (Fig. 5H and I; Table S4). We were unable to accurately determine the relative stability of CcnD, because it was extremely unstable in the WT strain following rifampin addition (data not shown).

10.1128/mBio.02385-21.4 TABLE S4 Half-life measurements. Download Table S4, XLSX file, 0.01 MB. Copyright © 2021 Sinha et al. 2021 Sinha et al. https://creativecommons.org/licenses/by/4.0/ This content is distributed under the terms of the Creative Commons Attribution 4.0 International license.

10.1128/mBio.02385-21.10 FIG S6 Northern blots corresponding to data presented in Fig. 5. (A) Representative Northern blots corresponding to CcnB, CcnC, and CcnD steady-state levels in the Δ*rny* mutant compared to a WT strain shown in Fig. 5G. (B to D) Representative Northern blots corresponding to CcnA, CcnB, CcnC, and CcnE stability curves shown in Fig. 5C, F, H, and I, respectively. Northern blot analysis was used to determine CcnA, CcnB, CcnC, and CcnE and 5S rRNA (loading control) amounts at the indicated time points following rifampin addition to the WT and the Δ*rny* mutant, which were growing exponentially as described in Materials and Methods. Download FIG S6, TIF file, 2.4 MB. Copyright © 2021 Sinha et al. 2021 Sinha et al. https://creativecommons.org/licenses/by/4.0/ This content is distributed under the terms of the Creative Commons Attribution 4.0 International license.

Finally, we investigated the role of RNase Y in Ccn-mediated *comC* target regulation. To this end, we constructed a translational fusion in which the 5′ untranslated region and the first 12 codons of *comC* are fused in-frame with the truncated E. coli β-galactosidase gene *lacZ*. The *comC*'-'*lacZ* translational fusion, driven from the constitutive vegetative promoter *vegT* (derived from the *vegII* promoter of Bacillus subtilis [Tables S1 and S2]), was integrated in the chromosomal *bgaA* locus in strain D39 (thereby knocking out pneumococcal β-galactosidase). Consistent with previous reports, deletion of all 5 Ccn sRNAs (Δ*ccnA*–*E*) relieved ComC translational repression and led to increased relative expression of β-galactosidase specific activity (∼3.5-fold) from *comC*'-'*lacZ* (Fig. 5J). Conversely, Δ*rny* led to decreased (∼3.8-fold) relative β-galactosidase specific activity from the *comC*'-'*lacZ* fusion (Fig. 5J), consistent with increased stabilization of Ccn sRNAs (Fig. 4) and increased translational repression of ComC. To further test this idea, we attempted to measure *comC*'-'*lacZ* expression in a Δ*rny* Δ*ccnA*–*E* mutant. Unexpectedly, the Δ*rny* Δ*ccnA*–*E* mutant exhibited a synthetic phenotype with severely impaired growth and low growth yield compared to the WT (data not shown). In contrast, a Δ*rny* Δ*ccnACDE* mutant did not exhibit a strong synthetic phenotype (data not shown). Relative expression of *comC*'-'*lacZ* is less elevated in the Δ*ccnACDE* than the Δ*ccnA*–*E* mutant and is reduced further in the Δ*rny* Δ*ccnACDE* mutant to near the WT level (Fig. 5J), consistent with stabilization of remaining CcnB in the Δ*rny* background. However, the transformation frequency (TF) of a Δ*rny* mutant was comparable to that of a WT strain (TF_Δ_*_rny_* = 0.38% versus TF_WT_ = 0.32%), using a spontaneous competence assay (Fig. S2B). Together, these results indicate that RNase Y-mediated regulation of Ccn sRNA stability has a consequential impact on Ccn-mediated target regulation in S. pneumoniae D39.

## DISCUSSION

**FIG 6:**
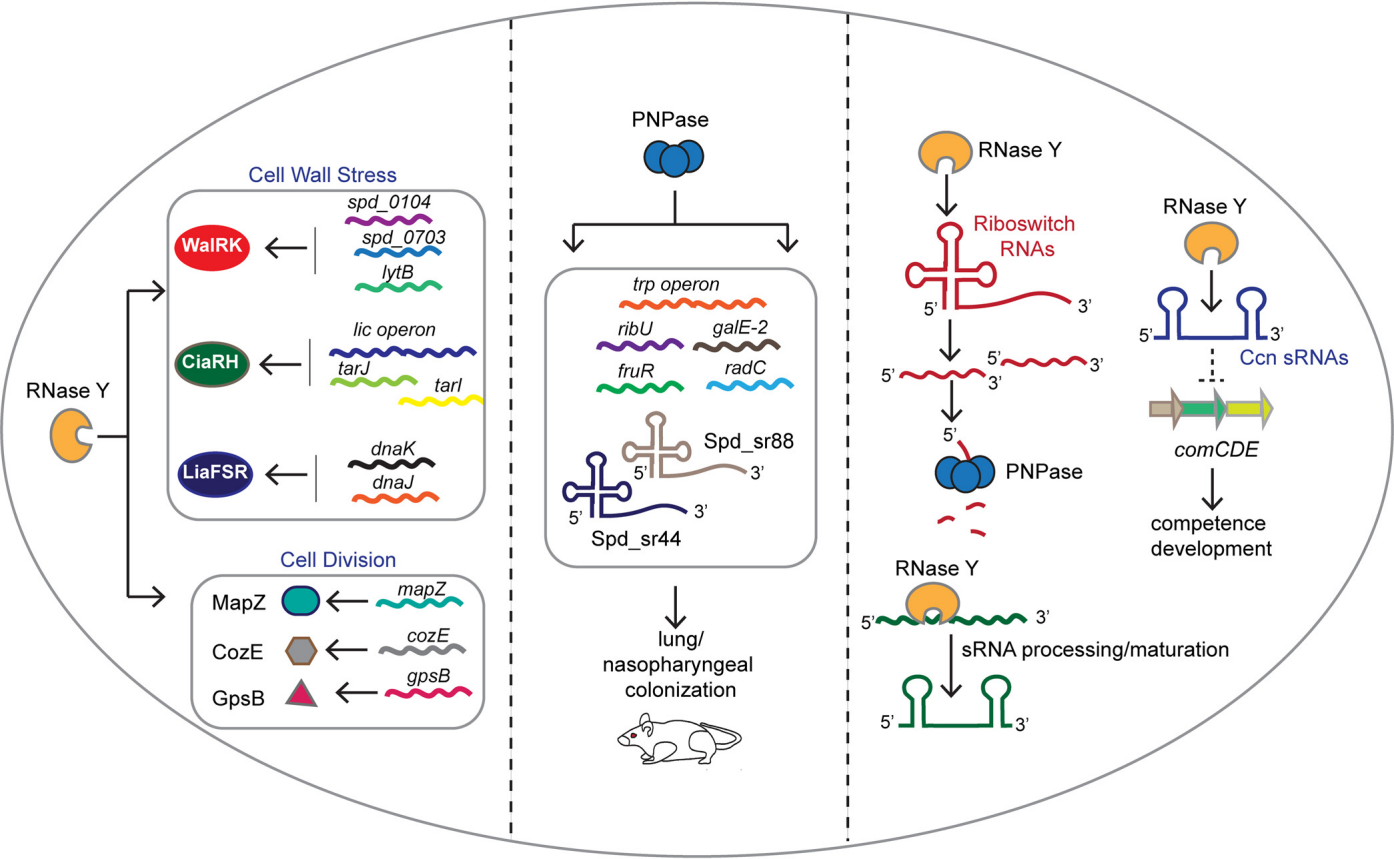
Summary of the major biological functions of RNase Y and PNPase. (Left) RNase Y regulates pneumococcal cell morphology by impacting transcripts encoding important cell division regulators. (Middle) PNPase mediates regulation of pneumococcal virulence gene expression. (Right) Roles of RNase Y and PNPase in regulatory RNA decay and processing.

This paper is the first report of the global roles of two highly conserved Gram-positive RNases, RNase Y and PNPase, in the human pathogen S. pneumoniae (summarized in Fig. 6). The loss of RNase Y significantly impacts gene expression by affecting ∼10% of the pneumococcal transcriptome and thereby causing pleiotropic phenotypes (Fig. 1A, C, and D; Fig. 2A and B; Fig. 3A and B; Tables 1 and 3). In contrast, PNPase exerts a relatively smaller impact on the transcriptome compared to RNase Y but, interestingly, regulates the expression of specific transcripts previously implicated in pneumococcal virulence control (Tables 2 and 4; Fig. 2C and D; Fig. 3C and D). Accordingly, the loss of PNPase severely attenuates S. pneumoniae virulence *in vivo* (Fig. 1E; Fig. S2A). This study also revealed that both RNase Y and PNPase work in concert to regulate the processing and decay of several regulatory RNAs, in particular, those characterized by the presence of 5′ *cis*-acting regulatory elements (Fig. 3B and D and 4; Fig. S3; Tables 2 and 4). In addition, RNase Y stabilizes the conserved pneumococcal *trans*-acting sRNAs CcnA to -E, further impacting Ccn-mediated target gene regulation (Fig. 5).

### RNase Y is a pleiotropic regulator in S. pneumoniae D39.

Deletion of *rny* in S. pneumoniae leads to a ∼2-fold increase in doubling time *in vitro* (Fig. 1A; Table S2), similar to prior observations with B. subtilis and C. perfringens (25, 27), and interferes with pneumococcal cell division (Fig. 1C). We identified several important pneumococcal cell wall and division regulators, including *mapZ* (encoding a midcell anchor protein), *cozE* (encoding a coordinator of zonal division), and *gpsB* (encoding a regulator that balances septal and elongation peptidoglycan synthesis), as being significantly upregulated in a Δ*rny* mutant (Fig. 2B; Table 1). In S. pneumoniae, MapZ guides tubulin-like FtsZ protein from midcell rings of dividing cells to the equators of new daughter cells (47, 48), whereas GpsB and CozE are major peptidoglycan (PG) biosynthesis regulators that play distinct but crucial roles at the midcell to maintain the normal ovococcus shape of pneumococcus by modulating the activities of different penicillin-binding proteins (PBPs), which catalyze peptide cross-link formation in peptidoglycan (49–51). Accordingly, Δ*mapZ* mutants exhibit a variety of abnormal cell shapes and sizes, decreased cell viability, increased doubling time, and aberrant FtsZ movement (47, 48, 52), while cells depleted of *gpsB* or *cozE* form elongated cells that are unable to divide or form chains that round up and lyse, respectively (49, 51, 53).

Several transcripts under the control of the essential TCS WalRK and the TCSs LiaFSR and CiaRH are impacted in the Δ*rny* mutant (Table 1), again consistent with cell wall and surface stress in cells lacking RNase Y, as numerous proteins in these regulons are known to impact cell morphology and chaining (54–58). In this regard, the defects in cell shape and morphology observed for B. subtilis Δ*rny* mutants were attributed to the upregulation of several PG biosynthesis genes, including *rodA* (27). It remains to be determined what cell wall stress is caused by absence of pneumococcal RNase Y and whether induction of certain proteins in these multiple surface stress TCS regulons can account for the defects in growth and morphology of the S. pneumoniae Δ*rny* mutant.

Besides responding to cell wall stress, the CiaRH TCS has been implicated in pneumococcal biofilm formation (59), competence (60), and virulence (61). In particular, the five conserved pneumococcal base-pairing sRNAs (CcnA to -E) negatively regulate translation of *comC*, which encodes the competence stimulatory peptide (42–44). We show here that RNase Y functions as a critical regulator of Ccn sRNA stability and impacts Ccn-mediated negative regulation of competence development in S. pneumoniae (Fig. 5). Interestingly, the recent Grad-seq analysis indicated possible stable RNA-protein complexes between the 3′-to-5′ exonuclease YhaM/Cbf1 and the Ccn sRNAs that were confirmed in pulldown experiments with Ccn sRNAs as bait in S. pneumoniae TIGR4 strain. In addition, CcnA to -E pulled down several degradosome components, including RNase J1/J2 and PNPase (23). The Gram-positive specific Cbf1 exonuclease has been implicated in trimming single-stranded RNA tails at the 3′ ends of Rho-independent terminated transcripts (16, 17, 23), thereby preventing decay by other exoribonucleases, such as PNPase and RNase R, that require an unstructured tail of 7 to 10 nucleotides (nt) for binding (17, 62). Although data presented here suggest that the Ccn sRNAs are targeted by RNase Y (Fig. 5), RNase Y was not identified as a strong Ccn sRNA interactor by Grad-seq (23), perhaps indicating complex dissociation during gradient centrifugation. Future experiments will determine whether Ccn sRNAs are direct substrates of RNase Y and whether Cbf1-mediated 3′ trimming impacts Ccn sRNA decay by RNase Y. Moreover, results in this paper raise the important question of whether RNase Y functions similarly to RNase E in mediating decay of *trans*-acting sRNAs that form sRNA-mRNA base pairs in S. pneumoniae and other Gram-positive bacteria.

Finally, lack of RNase Y affected the steady-state transcript levels of numerous key metabolic operons and known pneumococcal colonization and virulence factors, including *pavB* (fibronectin-binding protein and host interaction) (63), *clpL* (adaptor protein for ClpP protease) (64), CiaRH TCS regulon members (*licC*, *licB*, *and licA* [choline metabolism]) (65), LiaFSR TCS regulon members (*dnaK* and *dnaJ* [protein chaperones]) (66), WalRK TCS regulon members (*lytB* [glucosaminidase]), *spd_0104* (LysM-domain protein) (35), and PnpRS TCS regulon members (phosphate uptake) (35, 67) (Table 1). Thus, loss of RNase Y clearly exerts a global impact on the pneumococcal transcriptome that broadly affects physiology, growth, and virulence.

### PNPase is a key regulator of S. pneumoniae D39 virulence.

In contrast to the highly pleiotropic effects caused by the absence of RNase Y, the lack of PNPase minimally affected growth or morphology *in vitro*, but remarkably, caused strong attenuation *in vivo* (Fig. 1A, B, C, and E). The lack of phenotypes of the Δ*pnp* mutant *in vitro* may suggest that the pneumococcal 3′–5′ exoribonuclease RNase R can functionally bypass PNPase under certain experimental conditions. Notably, 10 of 20 protein-coding transcripts that were either upregulated (*ribU* [∼4-fold], *fruR* [∼2-fold], and *galE-2* [∼2-fold]) or down-regulated (*trpACDGE* [∼2.5- to 4-fold]) in the Δ*pnp* mutant included metabolic genes implicated in nasopharyngeal colonization and/or lung infection in a mouse model (35) (Fig. 2C and D; Table 2). The relative level of the full-length transcript of the T-box riboswitch Spd-sr88 located within the 5′ UTR of the *trp* operon (Fig. 4A) also decreased by ∼2-fold in the Δ*pnp* mutant, with concomitant accumulation of *spd-sr88*-derived decay intermediates (Fig. 4B; Table 4). These decay products are likely generated by RNase Y cleavage, since the relative full-length Spd-sr88 transcript levels increase by ∼11-fold in a Δ*rny* mutant (Fig. 4B; Fig. S5).

We do not yet know how PNPase positively regulates the *trp* operon in S. pneumoniae, but in general, *trp* operon regulation is important and complex in different bacteria and often involves RNA-based posttranscriptional mechanisms (68). For example, in B. subtilis under tryptophan-replete conditions, *trp* expression is repressed as a consequence of TRAP regulator protein-mediated transcription termination of the *trp* leader, which is subsequently degraded by RNase Y and/or J1 and PNPase. (69). In E. coli, tryptophan synthesis is regulated by a classical transcription attenuation mechanism, where under tryptophan-replete conditions, the upstream *trpL* leader peptide (TrpL) is translated efficiently, allowing formation of a terminator stem-loop that stops transcription before the downstream *trp* genes (70). Recently, the terminated *trpL* RNA generated by this attenuation mechanism was shown to function in Sinorhizobium meliloti as a base-pairing sRNA to destabilize several transcripts, including that of the *trp* biosynthesis genes (71). Likewise, previous studies in important Gram-positive pathogens, including Listeria monocytogenes and Enterococcus faecalis, show that terminated riboswitches are not “junk RNA” but function as mRNA- or protein-binding regulatory RNAs (72, 73). S. pneumoniae does not possess obvious homologs of TRAP or TrpL, but its *trp* operon instead contains the T-box (tRNA-sensing structure) riboswitch Spd-sr88. Whether the RNA decay products derived from *spd-sr88* (Fig. 4B) function as regulatory RNAs to destabilize the *trp* operon transcript in a Δ*pnp* mutant awaits further investigation. These combined results show that PNPase controls the transcript amounts of numerous genes required for pneumococcal pathogenesis, including the *trp* operon and the riboswitches Spd-sr88 and Spd-sr44 (Fig. 4B and E; Fig. S5; Tables 2 and 4) (35, 46), supporting the notion that PNPase is a key regulator of S. pneumoniae pathogenesis.

### RNase Y and PNPase play roles in sRNA processing and decay in S. pneumoniae D39.

Riboswitch turnover is important for recycling of the ligands to which they respond, and a role for RNase Y in this process was reported in B. subtilis (11, 74) and S. aureus (21). Here, we show that pneumococcal RNase Y mediates the initial endoribonucleolytic cleavage of 5′ *cis*-acting regulatory elements, which are subsequently degraded by PNPase. Likewise, in a Δ*pnp* mutant, we found that decay intermediates of eight riboswitch RNAs accumulated, while their corresponding full-length transcripts increased in abundance in the absence of RNase Y (Fig. 4; Fig. S3 and S5; Tables 3 and 4). These observations are consistent with a recent study in S. pyogenes showing that the coordinated actions of RNase Y and PNPase play a crucial role in the decay of riboswitches (20). In addition, our data indicate that RNase Y likely generates some sRNAs by cleaving larger transcripts, as observed for Spd-sr88 and Spd-sr116 (Fig. 4; Fig. S3 and S5). We conclude that RNase Y and PNPase work in tandem to degrade pneumococcal *cis*-acting regulatory RNAs, while RNase Y also plays an important role in sRNA processing and maturation. Whether RNase Y and PNPase interact together in a degradosome-like complex to impact regulatory RNA levels in S. pneumoniae will be resolved in future experiments.

## MATERIALS AND METHODS

### Bacterial strains and growth conditions.

Bacterial strains used in this study were derived from encapsulated S. pneumoniae serotype 2 strain D39W and are listed in Table S1. Strains were grown on plates containing Trypticase soy agar II (modified; Becton Dickinson [BD]) and 5% (vol/vol) defibrinated sheep blood (TSAII BA) at 37°C in an atmosphere of 5% CO_2_. Liquid cultures were grown statically in BD brain heart infusion (BHI) broth at 37°C in an atmosphere of 5% CO_2_. Bacteria were inoculated into BHI broth from frozen cultures or single colonies. For overnight cultures, strains were first inoculated into 17-mm-diameter polystyrene plastic tubes containing 5 ml of BHI broth and then serially diluted 100-fold into five tubes; these cultures were then grown for 10 to 16 h. Cultures with an OD_620_ of 0.1 to 0.4 were diluted to a starting OD_620_ between 0.002 and 0.005 in 5 ml of BHI broth in 16-mm glass tubes. Growth was monitored by measuring OD_620_ using a Spectronic 20 spectrophotometer. For antibiotic selections, TSAII BA plates and BHI cultures were supplemented with 250 μg/ml kanamycin or 150 μg/ml streptomycin.

### Construction and verification of mutants.

Mutant strains were constructed by transformation of competent S. pneumoniae strains with linear PCR amplicons as described previously (75). DNA amplicons containing antibiotic resistance markers were synthesized by overlapping fusion PCR. S. pneumoniae cells were induced to competence by the addition of synthetic competence stimulatory peptide 1 (CSP-1; Anaspec, Inc.). Markerless deletions and replacements of target genes were constructed using the Kan^r^ *rpsL*^+^ (Janus cassette) allele replacement method as described previously (76). In the first step, the Janus cassette was used to disrupt target genes in an *rpsL1* (Str^r^) strain background, and transformants were screened for kanamycin resistance and streptomycin sensitivity. In the second step, the Janus cassette was replaced by a PCR amplicon containing the desired mutation or replacement lacking antibiotic markers, and the resulting transformants were screened for streptomycin resistance and kanamycin sensitivity. Final transformants were isolated as single colonies three times on TSAII BA plates containing antibiotics listed in Table S1 and subsequently grown for storage in BHI containing the appropriate antibiotic. All constructs were confirmed by PCR amplification and sequencing.

### Microscopy.

After cultures reached an OD_620_ of ∼0.1 to 0.2, 1 ml was removed and centrifuged at 16,000 × *g* for 2 min at room temperature. Pellets were suspended in 50 μl of BHI broth. Cells were examined using either a Nikon E200 or a Leica DM 1000 LED phase-contrast microscope, and images were captured using a Nikon DS-Fi3 or a Leica ICC50W camera, respectively. A total of over 100 chains from each of two independent cultures of each strain were counted to determine distributions of numbers of cells per chain.

### RNA extraction.

RNA for high-throughput sequencing was prepared as described previously (77). Briefly, strains were grown in 30 ml of BHI starting at an OD_620_ of 0.002 in 50-ml conical tubes. RNA was extracted from exponentially growing cultures of IU3116 (wild-type parent; D39 *rpsL1* CEP::*kan rpsL^+^*) and its derived isogenic mutants IU5498 (D39 *rpsL1* Δ*pnp* CEP::*kan rpsL*^+^) and IU5504 (D39 *rpsL1 Δrny* CEP::*kan rpsL^+^*) at an OD_620_ of ∼0.1 from matched batches of BHI broth for mRNA-seq analysis or from IU1781 (wild-type parent; D39 *rpsL1*) and its derived markerless mutants IU4883 (D39 *rpsL1* Δ*pnp*) and NRD10092 (D39 *rpsL1* Δ*rny*) at an OD_620_ of ∼0.15 for sRNA-seq analysis using the FastRNA Pro Blue kit (MP Bio) according to the manufacturer’s guidelines. RNA extracted for mRNA-seq analysis was purified with an miRNeasy minikit (Qiagen), which included an on-column DNase I (Qiagen) treatment. For sRNA-seq analysis, RNA was alcohol precipitated following extraction and subsequently subjected to DNase treatment (Turbo DNase; Ambion) following the manufacturer’s protocol. Sample mixtures (total reaction volume of 50 μl) were incubated with Turbo DNase for 30 min at 37°C, and each reaction was stopped by addition of 150 μl of diethyl pyrocarbonate (DEPC)-treated water and 200 μl of neutral phenol–chloroform-isoamyl alcohol (Fisher). DNase-treated RNA samples were phenol extracted and alcohol precipitated. To isolate RNA for droplet digital PCR, RNA was extracted from exponential-growth-phase cultures following the procedure described above for sRNA-seq analysis. The amount and purity of all RNA samples isolated were assessed by NanoDrop spectroscopy (Thermo Fisher). RNA integrity of the samples used for RNA-seq library preparation was further assessed using the Agilent 2100 Bioanalyzer (Agilent Technologies).

### Library preparation and mRNA-seq.

cDNA libraries were prepared from total RNA by the University of Wisconsin—Madison Biotechnology Center as described previously (40). Briefly, total RNA was subjected to rRNA depletion using a RiboZero rRNA removal kit (Epicentre, Inc., Madison, WI, USA). Double-stranded cDNA synthesis was performed with rRNA-depleted mRNA using a ScriptSeq v2 RNA-seq library preparation kit (Epicentre, Inc., Madison, WI, USA) in accordance with the manufacturer’s standard protocol. The amplified libraries were purified using Agencourt AMPure XP beads. Quality and quantity were assessed using an Agilent DNA 1000 chip (Agilent Technologies, Inc., Santa Clara, CA, USA) and a Qubit double-stranded DNA (dsDNA) High Sensitivity assay kit (Invitrogen, Carlsbad, CA, USA), respectively. Libraries were standardized to 2 μM and cluster generation was performed using standard Cluster kits (v3) and Illumina Cluster Station. Single-end 100-bp sequencing was performed using standard SBS (sequencing by synthesis) chemistry (v3) on an Illumina HiSeq2000 sequencer. Images were analyzed using the standard Illumina pipeline, version 1.8.2.

### Library preparation and sRNA-seq.

sRNA libraries were prepared from total RNA as described previously (40). Briefly, 5 μg of DNase-treated total RNA was subjected to rRNA removal (RiboZero rRNA removal for Gram-positive bacteria; Illumina). rRNA-depleted samples were then subjected to RNA fragmentation using the Ambion RNA fragmentation kit (AM8740). Fragmented RNA was subjected to RNA 5′-polyphosphatase (Epicenter) treatment, which was performed to facilitate the 5′-adapter ligation step. Small RNA libraries were generated by Macrogen using the TruSeq small RNA library kit (Illumina). Then, 100-bp paired-end read sequencing was performed using an Illumina HiSeq2000 sequencer.

### RNA-seq analysis.

Raw sequencing reads from mRNA-seq were quality and adapter trimmed using Trimmomatic version 0.17 (78) with a minimum length of 90, while those corresponding to sRNA-seq were preprocessed for alignment with Cutadapt. The trimmed reads were mapped on the Streptococcus pneumoniae D39 (RefSeq NC_008533) genome and D39 plasmid pDP1 sequence (RefSeq NC_005022) using Bowtie2 (79). mRNA-seq and sRNA-seq analysis were performed as described previously using DESeq2 (77). Genes were defined as differentially expressed if their *P*_adj_ (*P* value adjusted for multiple testing) was <0.005. Primary data from mRNA-seq and sRNA-seq analyses was submitted to the NCBI Gene Expression Omnibus (GEO). The accession numbers for the sRNA-seq data corresponding to wild-type samples used for comparison of Δ*rny* and Δ*pnp* mutants are GSE148867 and GSE123437, respectively.

### ddPCR analysis.

One microgram of DNA-free RNA was reverse transcribed using random hexamers and Superscript III reverse transcriptase (RT) (Invitrogen) following the manufacturer’s protocol. For each sample, a no-RT (NRT) control reaction was performed. cDNA samples were diluted 1:10, 1:10^2^, 1:10^3^, or 1:10^6^, and 2 μl of each diluted RT and NRT PCR sample was added to a 22-μl reaction mixture containing 11 μl of QX200 ddPCR EvaGreen Supermix (Bio-Rad) and 1.1 μl of each ddPCR primer, each at 2 μM (Table S3). A single no-template control (NTC) for each ddPCR primer pair used in this study was included. Droplet generation from each reaction mixture was achieved via the QX200 automated droplet generator (Bio-Rad), and endpoint PCR was performed using a thermal cycler following the instructions from the manufacturer. A QX200 droplet reader (Bio-Rad) was used to analyze droplets from each individual reaction mixture, where PCR-positive and PCR-negative droplets were counted to provide absolute quantification of the target transcript. Data analysis was performed with QuantaSoft software (Bio-Rad), and the concentration of each target is expressed as copies per microliter. Reactions were performed using cDNA from at least three independent biological replicates, and transcript copies were normalized to 16S rRNA (internal control). Normalized transcript copy numbers were used to calculate fold changes of transcripts corresponding to target genes in different sets of mutants relative to the WT parent. Statistical analysis was performed using Student's *t* test in GraphPad Prism version 7.0.

### RNA stability assay.

To determine RNA stabilities, cultures were grown in BHI to exponential phase (OD_620_ ≈ 0.15) as described above, and a culture sample (0 h after the end of log-phase growth [*T*_0_]) was collected. Rifampin was added to inhibit transcription, and additional samples were collected 5, 10, 20, and 30 min after rifampin addition. All samples were subjected to hot phenol lysis as described previously (80). Briefly, 700 μl of sample was added to a mixture containing 800 μl of acid phenol–chloroform-isoamyl alcohol, pH 4.3 (Fisher Scientific), and 100 μl of lysis buffer (320 mM sodium acetate [pH 4.6], 8% [wt/vol] SDS, and 16 mM EDTA) equilibrated to 65°C. Samples were mixed at 65°C for 5 min and centrifuged for 30 min at 4°C to separate phases. The upper aqueous phase was extracted a second time with an equal volume of neutral phenol–chloroform-isoamyl alcohol, pH 6.7 (Fisher Scientific). RNA was ethanol precipitated and resuspended in DEPC-treated water. RNA concentration was measured using a NanoDrop 2000 (Thermo Fisher Scientific).

### Northern blot analysis.

Two micrograms of each RNA sample was loaded on 10% polyacrylamide gels containing 7 M urea or loaded onto 10% Criterion TBE-urea precast gels (Bio-Rad) and electrophoresed at 85 V. RNA samples were transferred to a Zeta-Probe GT membrane (Bio-Rad) using a Trans-Blot SD semidry transfer apparatus (Bio-Rad) following the manufacturer’s guidelines. Transferred RNA was UV cross-linked and hybridized overnight with 100 ng/ml of 5′ biotinylated DNA probe (Table S3) in Ultrahyb (Ambion) hybridization buffer at 42°C. Blots were developed using a BrightStar BioDetect kit protocol (Ambion), imaged with a ChemiDoc MP imager (Bio-Rad), and quantified using Image Lab software version 5.2.1 (Bio-Rad). Signal intensity corresponding to each sRNA was normalized to that of 5S rRNA, which served as an internal loading control. Decay curves corresponding to RNA stability time course experiments were generated by using GraphPad Prism version 7.0.

### Mouse models of infection.

All procedures were approved in advance by the Bloomington Institutional Animal Care and Use Committee (BIACUC) or UTHealth Animal Welfare Committee and were performed according to recommendations of the National Research Council. Experiments were performed as described in reference 76, with the following changes. Male ICR mice (21 to 24 g; Harlan) were anesthetized by inhaling 4% isoflurane (Butler Animal Health Supply) for 8 min. In two independent experiments, a total of 8 mice were intranasally inoculated with each bacterial strain to be tested. Bacteria were grown exponentially in BHI broth in an atmosphere of 5% CO_2_ to an OD_620_ of ∼0.1. Ten milliliters of culture was centrifuged for 5 min at 14,500 × *g* and then suspended in 1 ml 1× PBS to yield ∼10^7^ CFU ml^−1^. CFU counts were determined by serial dilution and plating. Fifty microliters of suspensions was administered intranasally as described previously (75). Mice were monitored visually at 4- to 8-h intervals, and moribund mice were euthanized by CO_2_ asphyxiation followed by cervical dislocation (IU-Bloomington), which was used as the time of death in statistical analyses. Alternatively, isoflurane-anesthetized moribund mice were euthanized by cardiac puncture-induced exsanguination followed by cervical dislocation (UTHealth). Kaplan-Meir survival curves and log-rank tests were generated using GraphPad Prism 7.0 software.

### β-Galactosidase assays.

Strains containing the *comC*'-'*lacZ* translational fusion were grown in BHI broth to exponential phase (OD_620_ ≈ 0.15). Samples were taken from each culture and assayed for β-galactosidase activity as described by Miller (81), with slight modifications. Briefly, 1 ml of culture was removed and centrifuged at 16,000 × g for 2 min at 4°C. Pellets were resuspended in 1 ml of Z-buffer containing 2-β-mercaptoethanol at a final concentration of 0.27%. Each sample mixture was lysed by subsequent incubation at 37°C for 10 min following the addition of 10 μl of 5% (vol/vol) Triton. One hundred microliters of lysed culture samples was then assayed for β-galactosidase specific activity as described previously (81).

### Competence assays.

Overnight cultures of strains were diluted into 5 ml of C+Y medium (casein-based medium supplemented with yeast extract), pH 8 (7), to a starting OD_620_ of ∼0.002. Starting from the initial inoculation and at 1-h intervals thereafter, 1 ml of a cell suspension was removed and mixed with 50 ng of amplicon DNA carrying a kanamycin resistance marker. After incubation for 90 min at 37°C in an atmosphere of 5% CO_2_, samples were serially diluted and plated on blood agar plates containing 250 μg/ml kanamycin and on blood agar plates without antibiotics to determine transformant CFU and total viable CFU, respectively. The transformation frequency (TF) was determined as the ratio of Kan^r^ CFU to total CFU per unit volume of cell suspension. Under these culture conditions, the natural transformation frequency of the wild-type strain followed a reproducible pattern with time in culture, with a high peak (2 × 10^−5^) about 2 h after inoculation (OD_620_ = 0.02 to 0.03). Accordingly, the natural transformation frequencies of the Δ*rny* mutant and wild-type strains were determined around an optical density of ∼0.02 to 0.03.

### Data availability.

The sRNA-seq data corresponding to Δ*rny* and Δ*pnp* mutants and the mRNA-seq data corresponding to all strains have been deposited in GEO under the accession number GSE173392.
