# Supplementary figures and images for: Pivotal Roles for Ribonucleases in Streptococcus pneumoniae Pathogenesis
Source: mBio. 2021 Sep 21;12(5):e02385-21. doi: 10.1128/mBio.02385-21 (PMC8546594; doi:10.1128/mBio.02385-21)

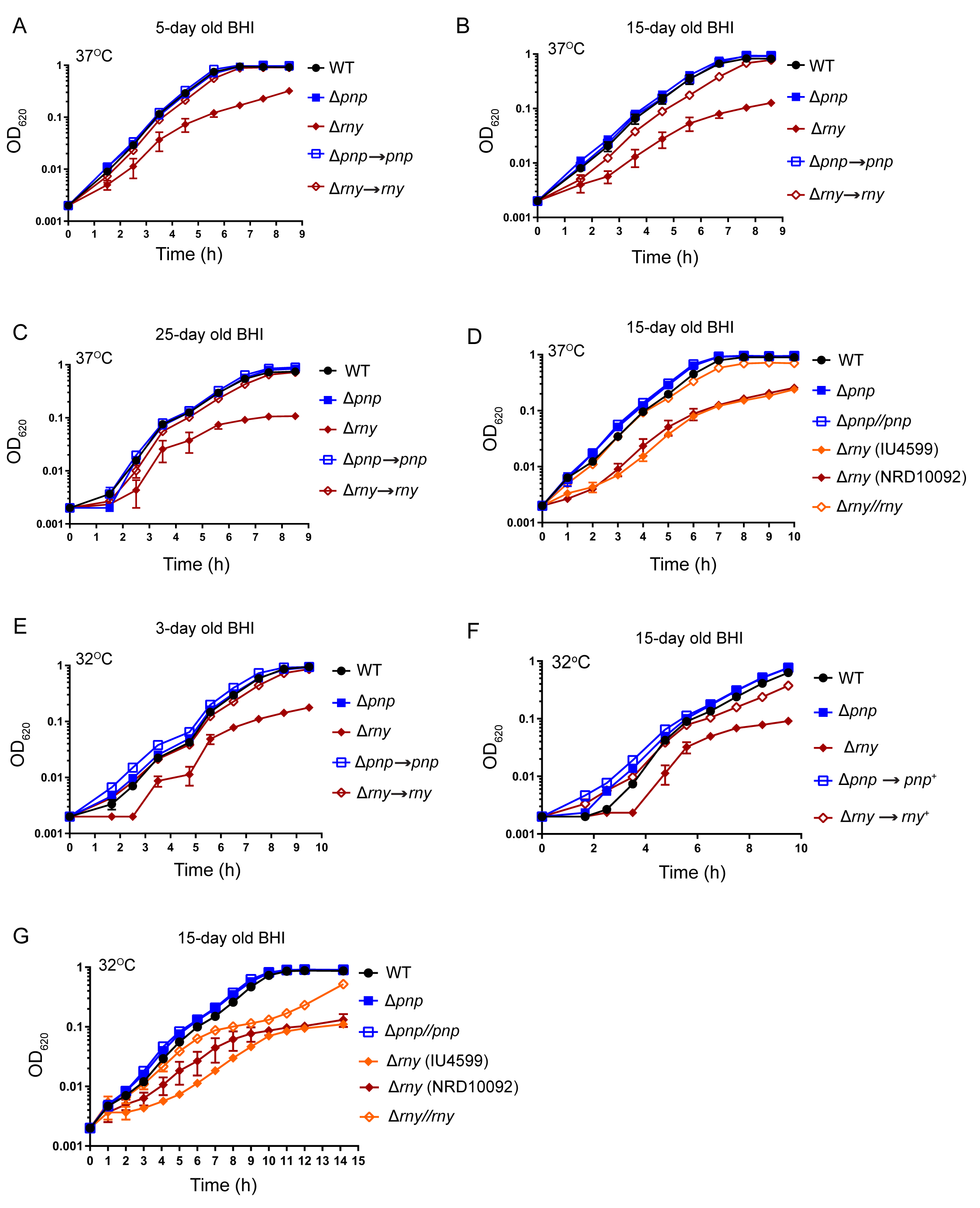

Supplement: FIG S1 [file mbio.02385-21-sf001.tif]

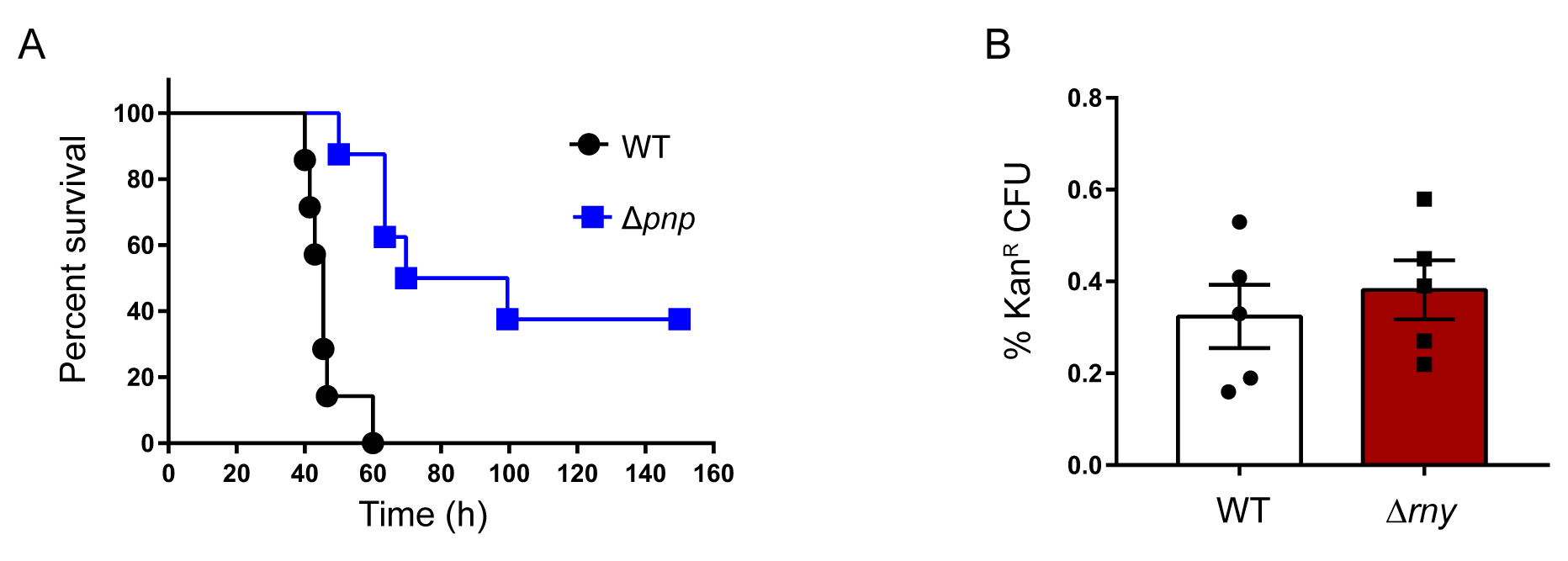

Supplement: FIG S2 [file mbio.02385-21-sf002.tif]

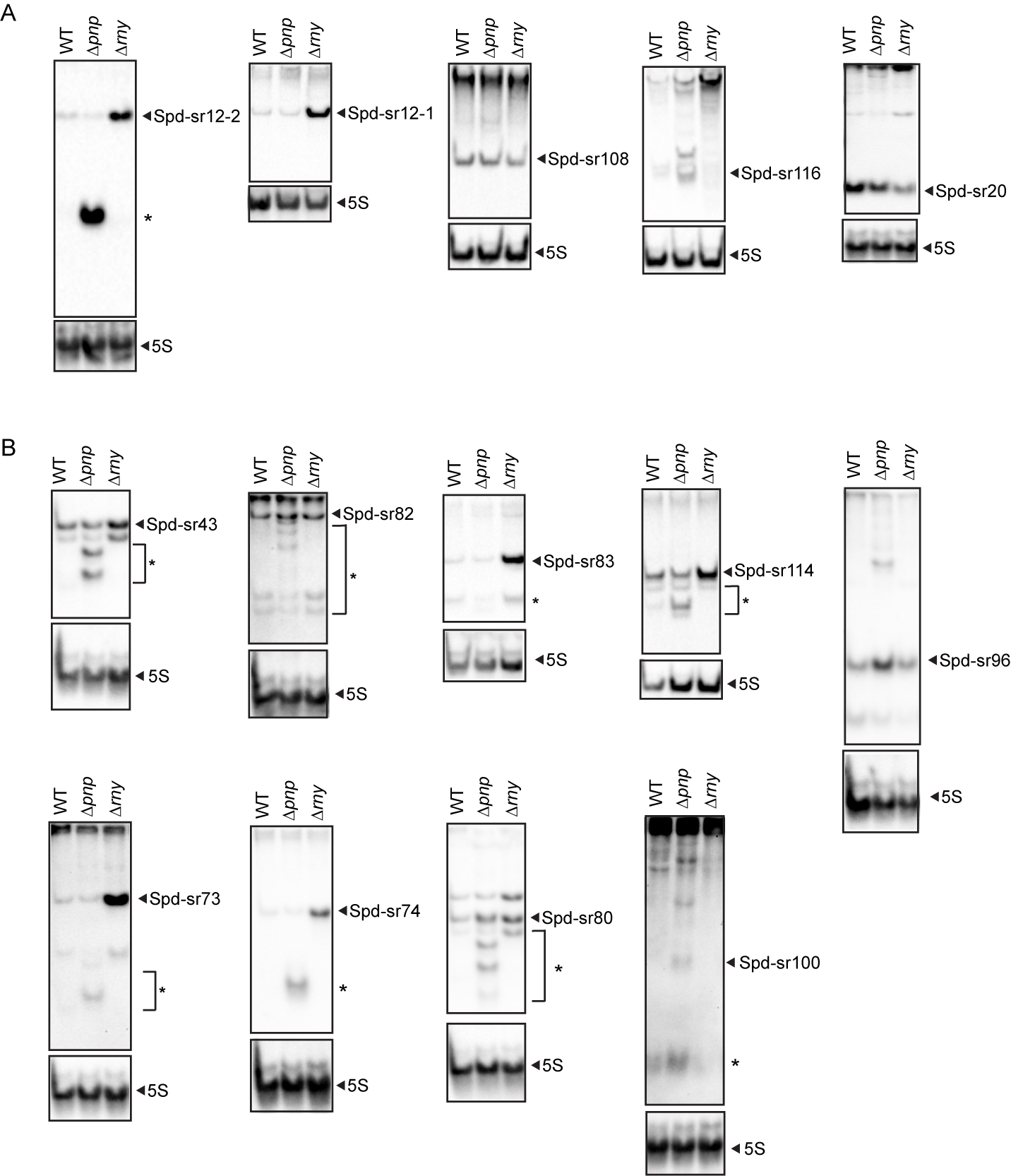

Supplement: FIG S3 [file mbio.02385-21-sf003.tif]

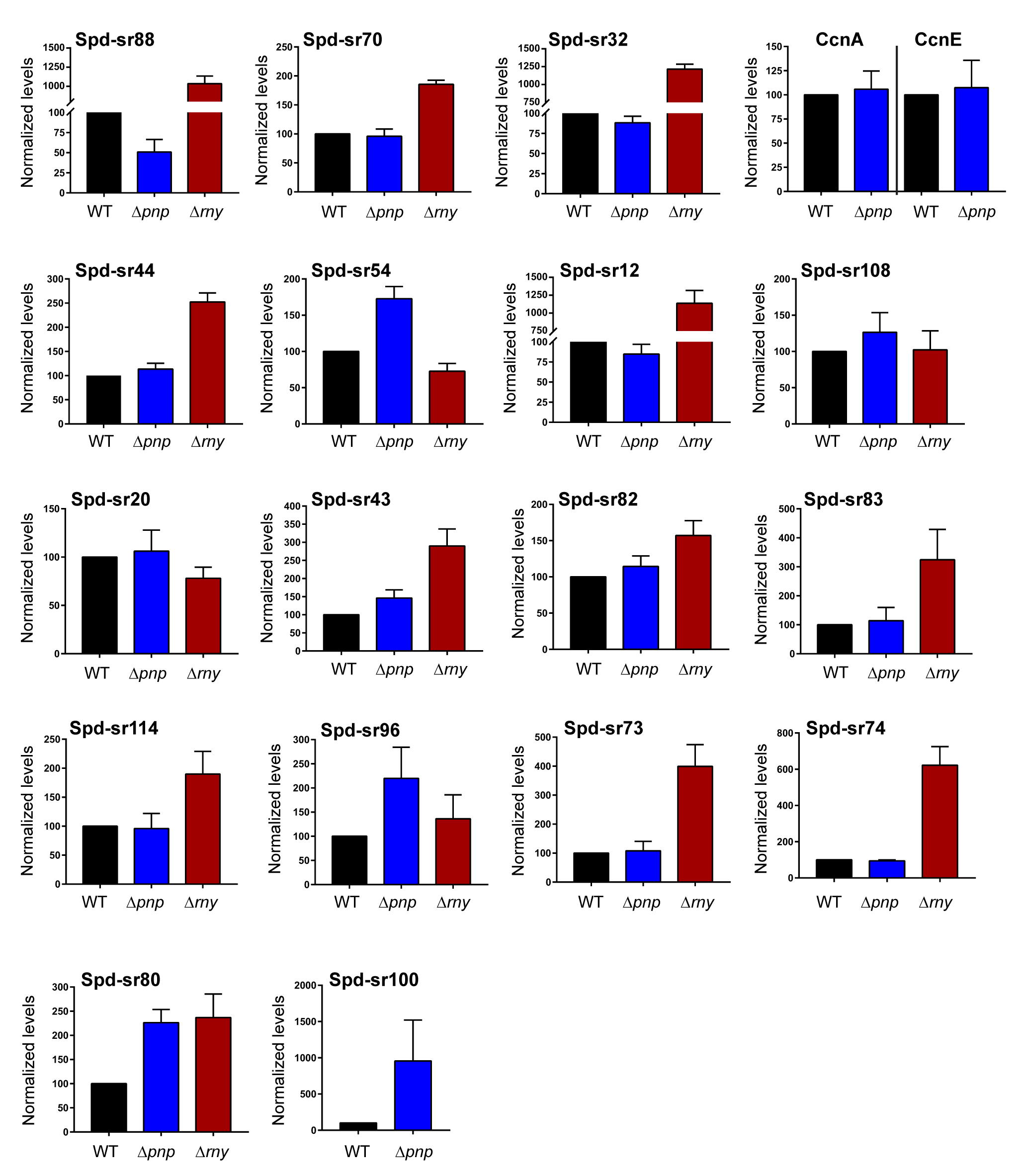

Supplement: FIG S5 [file mbio.02385-21-sf005.tif]

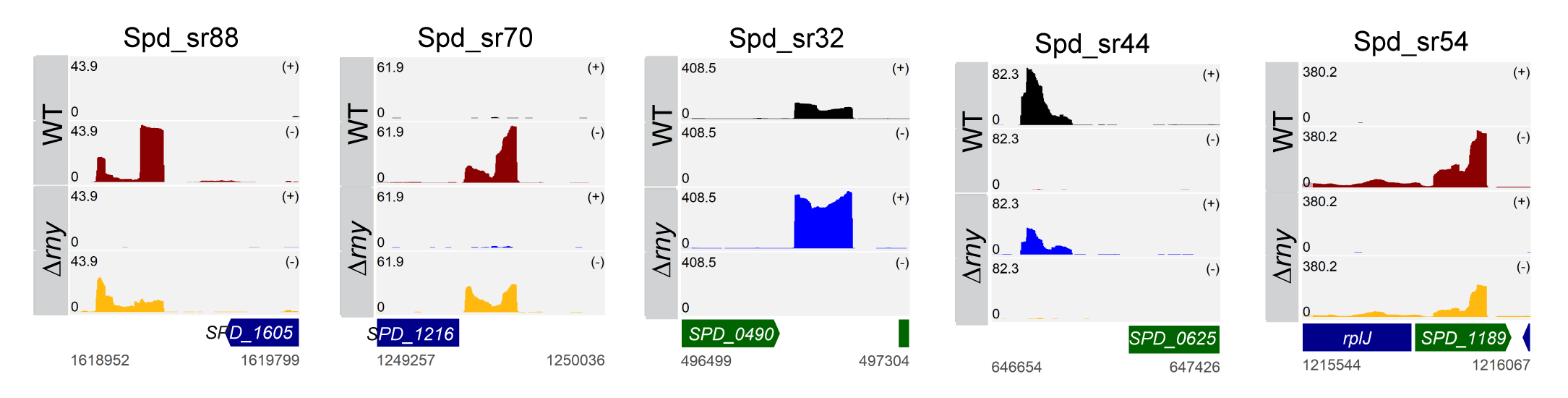

Supplement: FIG S4 [file mbio.02385-21-sf004.tif]

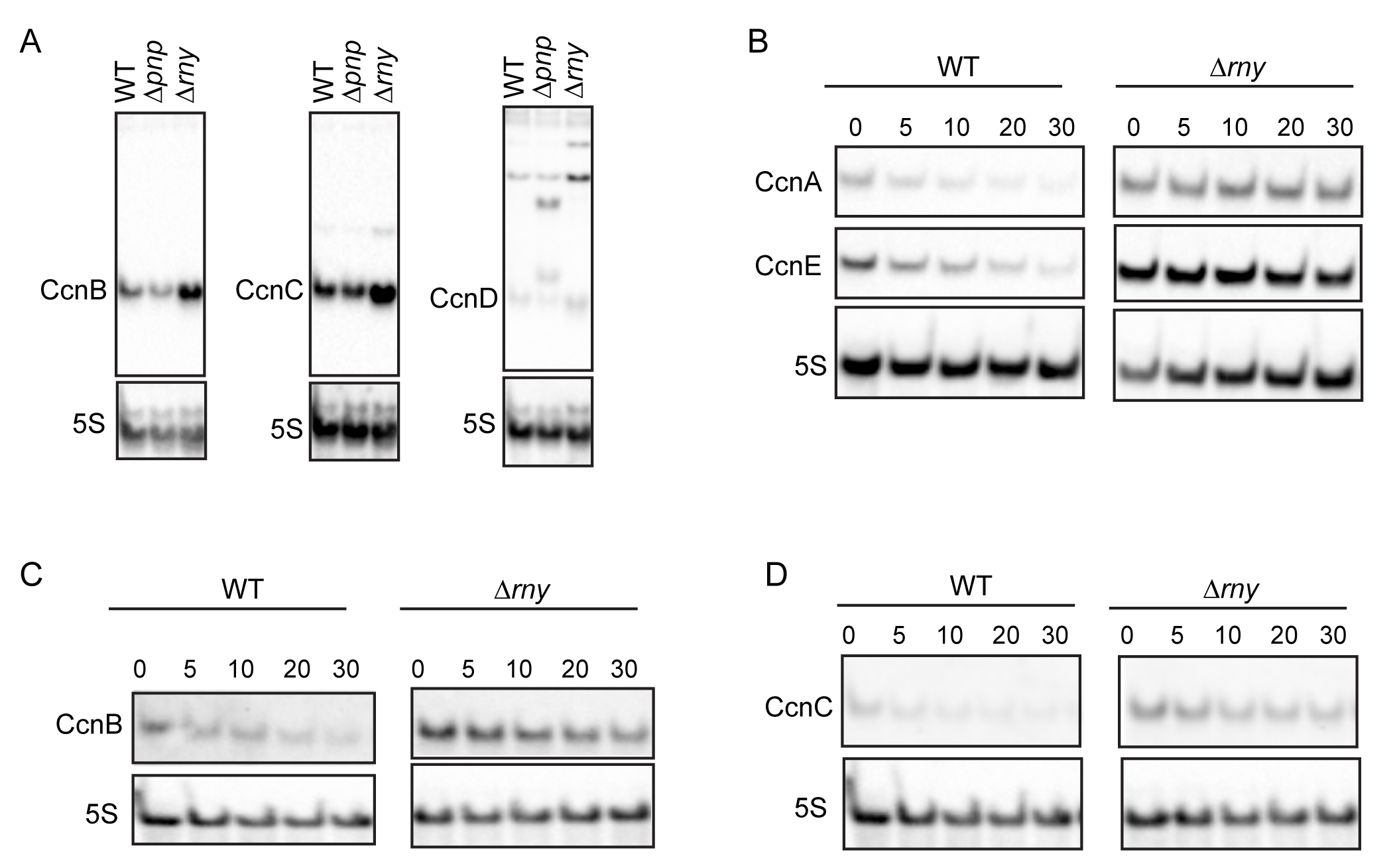

Supplement: FIG S6 [file mbio.02385-21-sf006.tif]
